# Virtual Reality for Pulmonary Rehabilitation: Comprehensive Review

**DOI:** 10.2196/47114

**Published:** 2023-10-02

**Authors:** Melpo Pittara, Maria Matsangidou, Constantinos S Pattichis

**Affiliations:** 1 Bernoulli Institute for Mathematics, Computer Science and Artificial Intelligence Groningen Netherlands; 2 CYENS—Centre of Excellence Nicosia Cyprus; 3 Department of Computer Science University of Cyprus Nicosia Cyprus; 4 Biomedical Engineering Research Centre University of Cyprus Nicosia Cyprus; 5 HealthXR Smart, Ubiquitous, and Participatory Technologies for Healthcare Innovation CYENS—Centre of Excellence Nicosia Cyprus

**Keywords:** breathing exercise, breathing exercise gaming, pulmonary rehabilitation, respiratory biofeedback, virtual reality

## Abstract

**Background:**

Pulmonary rehabilitation is a vital component of comprehensive care for patients with respiratory conditions, such as lung cancer, chronic obstructive pulmonary disease, and asthma, and those recovering from respiratory diseases like COVID-19. It aims to enhance patients’ functional ability and quality of life, and reduce symptoms, such as stress, anxiety, and chronic pain. Virtual reality is a novel technology that offers new opportunities for customized implementation and self-control of pulmonary rehabilitation through patient engagement.

**Objective:**

This review focused on all types of virtual reality technologies (nonimmersive, semi-immersive, and fully immersive) that witnessed significant development and were released in the field of pulmonary rehabilitation, including breathing exercises, biofeedback systems, virtual environments for exercise, and educational models.

**Methods:**

The review screened 7 electronic libraries from 2010 to 2023. The libraries were ACM Digital Library, Google Scholar, IEEE Xplore, MEDLINE, PubMed, Sage, and ScienceDirect. Thematic analysis was used as an additional methodology to classify our findings based on themes. The themes were virtual reality training, interaction, types of virtual environments, effectiveness, feasibility, design strategies, limitations, and future directions.

**Results:**

A total of 2319 articles were identified, and after a detailed screening process, 32 studies were reviewed. Based on the findings of all the studies that were reviewed (29 with a positive label and 3 with a neutral label), virtual reality can be an effective solution for pulmonary rehabilitation in patients with lung cancer, chronic obstructive pulmonary disease, and asthma, and in individuals and children who are dealing with mental health–related disorders, such as anxiety. The outcomes indicated that virtual reality is a reliable and feasible solution for pulmonary rehabilitation. Interventions can provide immersive experiences to patients and offer tailored and engaging rehabilitation that promotes improved functional outcomes of pulmonary rehabilitation, breathing body awareness, and relaxation breathing techniques.

**Conclusions:**

The identified studies on virtual reality in pulmonary rehabilitation showed that virtual reality holds great promise for improving the outcomes and experiences of patients. The immersive and interactive nature of virtual reality interventions offers a new dimension to traditional rehabilitation approaches, providing personalized exercises and addressing psychological well-being. However, additional research is needed to establish standardized protocols, identify the most effective strategies, and evaluate long-term benefits. As virtual reality technology continues to advance, it has the potential to revolutionize pulmonary rehabilitation and significantly improve the lives of patients with chronic lung diseases.

## Introduction

### Background

Pulmonary rehabilitation focuses on breathing exercises that can help people with chronic lung diseases improve lung function and reduce symptoms of chest tightness, chronic cough, and wheezing. Apart from lung diseases, pulmonary rehabilitation is commonly used for treatments related to hypertension, chronic pain, and cardiovascular disorders, such as coronary artery issues, arrhythmia, and myocardial infarction [1,2]. Breathing exercises may also offer effective and simple solutions for depressive and anxiety episodes, and other mental health–related disorders [3,4].

In recent years, virtual reality (VR) technology has been used in a wide variety of medical applications, including but not limited to areas involving the delivery of treatments for pulmonary diseases, such as asthma [5,6], chronic obstructive pulmonary disease (COPD), [7], and lung cancer [7], and mental health conditions, such as anxiety and stress-related disorders [5,8]. This is because exploration around physiological signals collected in VR can offer holistic breathing guidance options to users and provide them with breathing benefits [8-10]. Nowadays, individuals may explore and seek the assistance of professional coaches or use advanced devices that are specially designed for the purpose of breathing; however, these means often have a high cost and are time-consuming [5,7]. VR technology, on the other hand, is becoming one of the most accessible and low-cost solutions in the health care domain for breathing interventions [8,11]. Further, VR allows users to have full control over the environment they are exposed to. In combination with the use of biosensing technology, which provides acoustic, visual, and biofeedback guidance, VR users are offered the ability to consciously and self-effectively control and monitor their respiratory rate (RR) [2,5,12].

### COVID-19 and Pulmonary Rehabilitation

The novel coronavirus SARS-CoV-2 was identified in late 2019. A couple of months later, the World Health Organization (WHO) declared COVID-19 as a pandemic, as it affected 412,351,279 people (5,821,004 deaths) worldwide (February 13, 2022) [13]. The clinical symptoms in patients with COVID-19 included high fever, sore throat, cough, exhaustion, and dyspnea [13,14].

During the COVID-19 pandemic, patients’ medical care, including admission to clinics and use of emergency services, was affected owing to the danger of contamination and the limitations of medical service resources [14]. In this situation, clinical visits, nonurgent treatments, and nonearnest clinical issues, especially among vulnerable populations like people with pulmonary diseases, were initially interrupted and later resumed with a diminished scope [15]. 

Doctors had to confront the quandary of who could be treated at clinical centers or at home, or who could be allocated to the set number of beds in intensive care units [16]. New technologies helped support vulnerable populations during the pandemic, and VR helped overcome a variety of clinical challenges. This technology is quickly changing clinical training, patient therapies, and rehabilitation [14,15,17]. The pandemic has changed the clinical framework and placed standard methods with virtual telemedicine and software systems to provide clinical benefits for alleviating the effects of COVID-19 [18]. A significant part of the clinical framework involves rehabilitation, and it is significant owing to the pandemic period [19]. This might be driven by telehealth stages, as with the use of VR.

The fundamental objective of pulmonary rehabilitation is to further develop the patient’s psychophysical state [19]. Regardless of restricted admittance to hospitals for rehabilitation owing to COVID-19, VR technology can be applied to this group of patients. It can provide extensive help in various areas, including patient management and clinical treatment, monitoring of patient progression in rehabilitation or assessment of changes, and evaluation and advancement of body function, exercise, and consecutive participation [16,19]. Pulmonary rehabilitation is also beneficial after COVID-19 infection, even in patients who are recovering, who need assisted ventilation or oxygen therapy [20]. Additionally, COVID-19 survivors experience stress, depression, and low quality of life, and the symptoms of dyspnea and fatigue can last more than 3 months after infection. Recent evidence has shown that pulmonary rehabilitation can alleviate these symptoms and can improve exercise performance, lung function, and quality of life in COVID-19 patients and survivors [21-23].

However, scientific studies examining and evaluating the opportunities and challenges of VR for breathing remain limited. In this literature review, we introduce state-of-the-art VR technologies relevant to pulmonary rehabilitation and breathing exercises by analyzing recent related articles on the subject. Owing to the lack of studies related to breathing gaming exercises, for this review, we only examined and evaluated 32 available studies from the last decade [5-8,10,24-50]. Evidence from related empirical and experimental studies that comprised several types of breathing exercises and patterns was systematically reviewed to address the following research questions:

- Is VR an effective solution for breathing exercises?
- Which are the most common VR contents used for breathing exercises in gaming?
- How feasible is gamified biofeedback breathing VR for real-world deployment?
- What are the current barriers to biofeedback VR technologies?
- What are the future directions of biofeedback VR technologies?

## Methods

### Design

This review was conducted according to Bargas-Avila and Hornbæk [51] and the Cochrane methodology [52,53], which involved 5 phases. The phases are described below.

### Procedure

#### Phase 1: Detailed Assessment of Publications

Electronic libraries: The research was conducted with the use of 7 electronic libraries, which cover a balanced choice of multidisciplinary sources. The libraries were as follows: (1) ACM Digital Library (ACM), (2) Google Scholar, (3) IEEE Xplore (IEEE), (4) MEDLINE, (5) PubMed, (6) Sage, and (7) ScienceDirect (SD). The search was delimited to a timeframe of 13 years (2010 to 2023).

Search terms: The following 3 queries were used in all the libraries since the aim was to cover any type of VR technology for breathing:

1. Virtual Reality AND Breath
2. Virtual Reality AND Pulmonary Rehabilitation
3. Virtual Reality AND Breathing games

Search procedure: The search terms were used to examine the publication’s title, abstract, and keywords.

Search results: The search results in Phase 1 can be seen in Table 1.

**Table 1:** Search results (N=2319).

Search terms	ACM^a^ (n=524)	Google Scholar (n=678)	IEEE (n=13)	MEDLINE (n=293)	PubMed (n=67)	Sage (n=362)	SD^b^ (n=382)
Virtual Reality AND Breath	133	293	11	224	58	162	220
Virtual Reality AND Pulmonary Rehabilitation	220	237	0	32	8	96	77
Virtual Reality AND Breathing games	171	148	2	37	1	104	85

^a^ACM: ACM Digital Library.

^b^SD: ScienceDirect.

#### Phase 2: Publications Retrieved for Detailed Evaluation

First exclusion: All search results from Phase 1 were imported into Mendeley electronic library. Possible entries with wrong years were excluded (625 wrong-year entries were removed). This elimination decreased the number of papers to 1694.

Second exclusion: Duplicate papers, either extracted from one or more libraries, which either produced or concluded the same outcome, were removed from this review. Moreover, duplicate papers, extracted from one or more similar terms, which either produced or concluded the same outcome, were also removed. A total of 375 duplicate publications were removed, leaving 1319 different papers.

Third exclusion: The entries were narrowed down to original full papers that were written in English. We excluded papers that did not have the full text available (ie, we did not have access to the full text) and papers that were not original full papers, such as workshops, posters, speeches, reviews, magazine articles, and generally grey literature without formal peer review. As a result, 312 papers were excluded, leaving 1007 papers.

#### Phase 3: Final Exclusion

**Figure 1:**
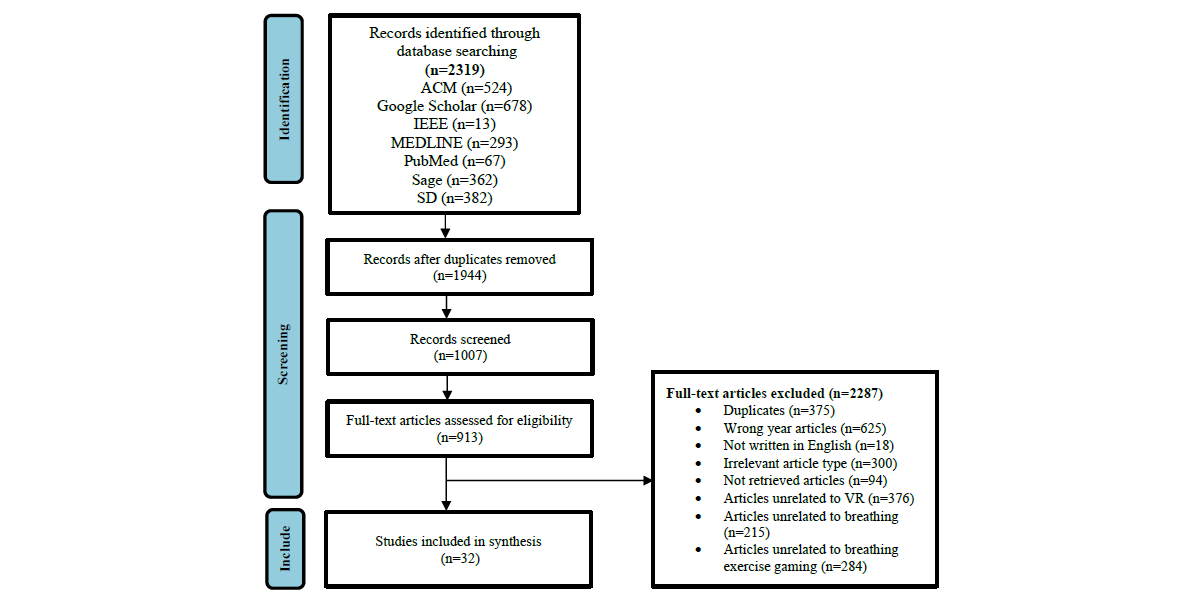
PRISMA (Preferred Reporting Items for Systematic Reviews and Meta-Analyses) flowchart of the literature review. ACM: ACM Digital Library; SD: ScienceDirect; VR: virtual reality.

Since this review was focused on VR technologies related to breath and breathing exercise gaming, we excluded papers that examined and used other types of technologies that were not related to VR or to breathing exercise gaming. Moreover, research that was only related to breathing without the use of VR was excluded. In this phase and based on these conditions, any irrelevant studies that appeared in Phase 1 and were not removed in Phase 2 were excluded. However, these studies may appear in our findings since relevant words were contained in our research but did not match the specific technical content. Based on these conditions, we removed 376 studies unrelated to VR, 215 studies unrelated to breathing, and 284 studies unrelated to breathing exercise gaming. We ended up with 32 relevant papers (24 journal articles and 8 conference papers; there were no book chapters). The flowchart is presented in Figure 1. All relevant studies were downloaded for examination.

#### Phase 4: Data Gathering

All related information from the studies was extracted for examination. An Excel file was created, and the following data were extracted from each paper: sample size of the population studied, methodology, instruments, apparatus, VR content, VR interventions, types of biofeedback sensors, types of biosignals, VR feasibility, key findings, current VR limitations, and VR future directions. Moreover, we categorized each study based on the results as positive (+), negative (−), or neutral.

#### Phase 5: Data Analysis

The data gathered in Phase 4 were analyzed using descriptive statistics. Consequently, the literature was reviewed to support and enhance the additional knowledge provided by the study. Thematic analysis was used as an additional methodology to classify our findings based on themes. The following themes were considered: VR training, VR interaction, types of virtual environments (VEs), VR effectiveness, VR feasibility, VR design strategies, VR limitations, and VR future directions. Intercoder reliability was assessed between the researcher and research assistant. The Cohen kappa formula was used to calculate the similarity between the researcher and research assistant, and the similarity value was 0.89.

## Results

### Objectives, Study Design, and Interventions

The search identified 32 studies related to the effective use of VR for breathing exercises. The sample, study design, objective, and intervention of each study are presented in Table 2. Of the 32 studies, 24 involved healthy individuals [6,8,10,24,26,28-38,40,41,43-48], 1 involved individuals at high risk of developing anxiety disorders [39], 1 involved patients with lung cancer [7], 1 involved patients with pneumonia [49], 2 involved patients with COPD [27,42], 2 involved patients with COVID-19 [49,50], and 1 involved patients with asthma [5]. The breathing exercises were mostly related to breathing therapy (9/32) [5,6,10,25,27,33,48,49,54] and anxiety management (13/32) [8,28,29,32,34,36,37,39-41,43-45]. Most of the reviewed papers based their research on pilot (16/32) or control studies (11/32), while some of the papers (4/32) only described the design and development processes of their systems. Most of the systems (24/32) were designed based on breathing patterns and techniques related to the needs of the populations. For example, for participants who had lung cancer, the VR environments were created for them to perform normal breathing exercises (ie, 10-20 breaths per min) [7], as opposed to the environments for those who had pneumonia or COVID-19 [49], and the VR environments were created to navigate the participants’ breathing into a positive expiratory pressure technique (ie, involving breathing with an expiratory resistance that allows air to flow freely when inhaling but harder due to resistance when exhaling) [8]. A different example was provided in a study that included participants who had COPD, and the VR system offered them an avatar assistant, who presented educational content and physical exercises [27].

**Table 2:** Virtual reality breathing studies: sample, study design, objective, and intervention.

Study	Sample	Study design	Objective	Intervention
Abushakra et al [7], 2014	125 males and females; age not reported	N/A^a^	Develop breathing therapy	Normal breathing with visual feedback
Betka et al [49], 2022	19 males and 7 females; age: 18-55 years	CS^b^ (mixed study), randomized, single-blind cross over	Investigate the effect of VR^c^ on COVID-19 patients’ rehabilitation	Normal breathing with audiovisual feedback
Blum et al [25], 2019	29 males and 31 females; average age: 33.5 years	CS (VR, non-VR)	Investigate the effect of VR and slow-paced breathing on HRV^d^	Slow breathing with visual feedback
Blum et al [10], 2020	16 males and 56 females; age: 18-49 years	CS	Develop VR biofeedback system–based respiratory treatment	Slow diaphragmatic breathing with visual feedback
Brammer et al [41], 2021	9 males and females; age not reported	PS^e^	Develop VR biofeedback scenarios for stress-exposure training	Slow breathing with visual feedback
Charoensook et al [31], 2019	21 males and females; age: 20-24 years	CS (VR, non-VR)	Investigate how VR systems affect HR^f^ and RR^g^	Normal breathing with visual feedback
van Delden et al [5], 2020	8 males and 4 females; age: 6-8 years	PS	Develop VR systems based on spirometry for children with asthma	Spirometry test with visual feedback
Feinberg et al [47], 2022	21 males and females; age: 18-34 years	Mixed methods	Design a VR system for meditation	Normal, slow, and diaphragmatic breathing with visual feedback
Gummidela et al [45], 2022	16 males and 14 females; age: 18-35 years	CS (game, nongame)	Develop a VR system for breathing training	Deep breathing with visual feedback
Heng et al [43], 2020	9 participants; gender and age not reported	PS	Integrate a breathing sensor with a VR system for pulmonary rehabilitation	Several breathing techniques with visual feedback
Hu et al [35], 2021	3 males and 3 females; age: 5-8 years	PS	Examine the effectiveness of combining breathing exercises with music rhythm through VR	Several breathing techniques with visual feedback and musical guidance
Jung et al [27], 2020	6 males and 4 females; age: 63-75 years	PS	Pulmonary rehabilitation among participants with COPD^h^	Educational videos and physical exercises with audiovisual feedback
Kluge et al [36], 2021	13 males and 17 females; age: 22-39 years	PS	Develop a VR system for stress management training	Several breathing techniques with visual feedback
Ladakis et al [37], 2021	2 males and 2 females; age: 23-59 years	CS (VR, non-VR)	Examine stress reduction in work environments through VR	Deep breathing exercises with visual feedback
Mevlevioğlu et al [38], 2021	7 males and 4 females; age: 18-30 years	PS	Develop a VR system for height exposure therapy (acrophobia)	Natural breathing with visual respiratory feedback
Michela et al [44], 2022	9 males; age: 26-55 years	PS	Develop a VR system for police officers’ breathing performance	Deep and slow diaphragmatic breathing with visual feedback
Patibanda et al [34], 2017	16 males and 16 females; age not reported	PS	Develop a VR system for PLB^i^ training	PLB with visual feedback
Prpa et al [26], 2018	4 males and 7 females; age: 24-44 years	PS	Investigate breathing patterns according to VR to enhance breath awareness	Slow breathing with visual feedback
Quintero et al [32], 2019	5 males and 6 females; age: 23-32 years	PS	Develop a VR application for HRV analysis	Normal and slow breathing with visual feedback
Rockstroh et al [39], 2021	16 males and 29 females; age: 19-52 years	DD^j^	Develop a mobile VR-based respiratory biofeedback system	Diaphragmatic breathing with visual feedback
Rodrigues et al [50], 2022	22 males and 22 females; age: 18-80 years	CS	Investigate a VR system for the sensation of dyspnea in COVID-19 patients	Not reported
van Rooij et al [40], 2016	52 males and 34 females; age: 8-12 years	PS	Develop a VR breathing system for anxiety in children	Diaphragmatic breathing with visual feedback
Rutkowski et al [42], 2021	9 males and 41 females; age: 45-85 years	CS (VR, non-VR)	Examine the effectiveness of VR for depression and anxiety in participants with COPD	Pulmonary rehabilitation exercises with visual feedback
Shih et al [24], 2019	12 males and 31 females; average age: 25.9 years	PS	Develop a mobile app for detecting breathing phases	Normal and slow-paced breathing
Soyka et al [28], 2016	11 males and 10 females; age: 20-45 years	CS	Develop a VR application for stress management	Paced breathing techniques with visual feedback
Desnoyers-Stewart et al [33], 2019	Not reported	DD	Develop a VR application for breathing synchronization between participants	Paced breathing with visual feedback
Tabor et al [8], 2020	N/A	DD	Develop a VR breathing exercise system to support pneumonia rehabilitation	Positive expiratory pressure with visual feedback
Tao et al [46], 2020	3 participants; gender and age not reported	PS	Examine the latent breath input for music instrument performance in VR	Normal breathing with visual and audio feedback
Tatzgern et al [48], 2022	10 males and 2 females; age not reported	DD	Develop a VR system for resistance breathing training	Several breathing techniques with visual feedback
Tinga et al [29], 2018	23 males and 37 females; age: 18-31 years	CS	Examine the effectiveness of respiratory biofeedback through VR meditation	Audio-guided meditation through visual feedback
Tu et al [6], 2020	10 males and females; age not reported	PS	Develop a VR system for breathing training at home	RSA^k^ with visual feedback
Zafar et al [30], 2018	100 males; average age: 23 years	CS	Develop a VR system for breathing training	Paced breathing with visual feedback

^a^N/A: not applicable.

^b^CS: control study.

^c^VR: virtual reality.

^d^HVR: heart rate variability.

^e^PS: pilot study.

^f^HR: heart rate.

^g^RR: respiratory rate.

^h^COPD: chronic obstructive pulmonary disease.

^i^PLB: pursed-lip breathing.

^j^DD: design and development study.

^k^RSA: respiratory sinus arrhythmia.

Various studies involving the treatment of anxiety disorders decided to choose healthy participants for the needs of the experimentations. In particular, among 15 studies, healthy individuals tried several VEs to perform slow (ie, 4-10 breaths per min) [8,10,25,26,41,44,45], normal [7,8,31,32,48], and diaphragmatic breathing exercises (ie, inhale through the nose moving the air toward the lower belly and exhale through the mouth) [39,40,46]. Moreover, healthy individuals participated in studies where VEs were created to guide them on breathing patterns like respiratory sinus arrhythmia (ie, synchronization between heart rate variability [HRV] and RR) [6] and pursed-lip breathing (ie, inhale through the nose with the mouth closed and exhale through tightly pressed lips) [38]. Finally, few studies examined the effectiveness of VR in children for treating asthma by practicing breathing exercises with the use of spirometry [5] and anxiety by practicing diaphragmatic breathing [40].

### Instruments

#### Self-Reported Data

As mentioned above, most of the studies enhanced conventional breathing training through VR interventions. To evaluate the effectiveness of the VR system, studies used several measures, which are presented in Table 3. All the reviewed studies (32/32) collected participants’ demographic information (eg, age, sex, ethnicity, educational level, etc). Half of the reviewed studies (14/32) examined the effect of VR breathing training on mental health (ie, anxiety) [6,25,26,28-30,32,34,36,37,39-41].

**Table 3:** Virtual reality breathing studies: measures, training, and study duration.

Study	Measures	Training	Study duration
Abushakra et al [7], 2014	Physiological data: Lung size, lung capacity, and total lung capacity	No	Not applicable
Betka et al [49], 2022	Quantitative data: GAD-7^a^ Physiological data: RR^b^, respiratory rate variability, HR^c^, and SpO2	No	VRE^d^: 5 min; FES^e^: 16 min
Blum et al [25], 2019	Quantitative data: VAS^f^, STAI^g^, Cognitive Interference Questionnaire, State Mindfulness Scale, relaxation self-efficacy, mind wandering, and respiration exercise experience Physiological data: HR	Yes	VRE: not reported; FES: 10 min
Blum et al [10], 2020	Quantitative data: User Experience Questionnaire Physiological data: HR and respiratory sinus arrhythmia	No	VRE: 7 min; FES: not reported
Brammer et al [41], 2021	Physiological data: RR	Yes	VRE: 15 min; FES: 15 min × 10 sessions, 3 weeks
Charoensook et al [31], 2019	Quantitative data: Self-perceived fitness level, gaming experience, and VR^h^ experience Physiological data: HR and RR	No	VRE: 15 min; FES: 2 h
van Delden et al [5], 2020	Qualitative data: Questionnaire about what happened in the game, what was liked the most, and what was liked the least Quantitative data: VAS Physiological data: Lung function	No	VRE: not reported; FES: 30 min × 4 sessions
Feinberg et al [47], 2022	Qualitative data: Expert meditator interview and qualitative learning assessment (quality of experience) Quantitative data: Quantitative learning assessment	No	VRE: 5-15 min; FES: 10 sessions × 25 min
Gummidela et al [45], 2022	Quantitative data: Stroop Color and World Test Physiological data: RR	Yes	VRE: 5 min; FES: 20 min × 6 sessions
Heng et al [43], 2020	Quantitative data: Game experience Physiological data: Strong breathing, long breathing, and breathing strength control	No	VRE: not reported; FES: not reported
Hu et al [35], 2021	Quantitative data: Game experience Physiological data: Breathing strength	Yes	VRE: 6 min; FES: 23 min
Jung et al [27], 2020	Quantitative data: Patient Activation Measure, GAD-7, Patient Health Questionnaire-9, Short Physical Performance Battery, and Edmonton Frail Scale. Physiological data: HR and oxygen saturation	No	VRE: 20 min per day × 8 weeks; FES: 75 min
Kluge et al [36], 2021	Quantitative data: Nijmegen questionnaire Physiological data: RR	Yes	VRE: 9 min; FES: 90 min
Ladakis et al [37], 2021	Quantitative data: Self-reports and User Experience Questionnaire Physiological data: HR and electrodermal signal	No	VRE: 2.5 min; FES: 27 min
Mevlevioğlu et al [38], 2021	Quantitative data: Igroup Presence Questionnaire and visual height intolerance scale Physiological data: HR, brain electrical activity, electrodermal signal, and RR	No	VRE: 5 min; FES: not reported
Michela et al [44], 2022	Quantitative data: Dutch STAI, prior gaming experience, short self-constructed questionnaire before and after the intervention, and IMI^i^ Physiological data: HRV^j^	Yes	VRE: 15 min; FES: 10 sessions × 15 min, 4 weeks
Patibanda et al [34], 2017	Qualitative data: Formal Analysis of Gameplay Physiological data: RR	Yes	VRE: 3 min; FES: not reported
Prpa et al [26], 2018	Qualitative data: Interviews Quantitative data: Music emotion recognition Physiological data: RR	Yes	VRE: 6 min; FES: 45 min
Quintero et al [32], 2019	Quantitative data: Customized questionnaire Physiological data: HRV	No	VRE: not reported; FES: 30 min
Rockstroh et al [39], 2021	Quantitative data: Breath awareness scale, PSS-10^k^, and Copenhagen Burnout Inventory Physiological data: RR, inhalation duration, and exhalation duration	Yes	VRE: 8 min; FES: 46 min
Rodrigues et al [50], 2022	Quantitative data: Edmonton Symptom Rating Scale, Borg Scale, HADS^l^, and Mini-Mental State Examination Physiological data: HR, RR, blood pressure, and SpO2	No	VRE: 10 min; FES: 40 min
van Rooij et al [40], 2016	Qualitative data: Qualitative observations of participants’ behavior Quantitative data: STAI, 7-point Likert scale on the experience of playing, self-reported positive and negative affect, and IMI Physiological data: diaphragm expansion and RR	No	VRE: 7 min; FES: not reported
Rutkowski et al [42], 2021	Quantitative data: Perception of Stress Questionnaire and HADS Physiological data: Lung function and expiratory volume	No	VRE: 20 minutes × 5 times, 2 weeks; FES: 30 min
Shih et al [24], 2019	Quantitative data: Self-reports based on 7-point Likert scales Physiological data: Acoustic signal of respiration, inhalation duration, exhalation duration, breathing cycles, and HRV	Yes	VRE: 6 min; FES: 50 min
Soyka et al [28], 2016	Quantitative data: PSS-10 Physiological data: RR, HR, HRV, and blood pressure	No	VRE: 10 min; FES: 25 min
Desnoyers-Stewart et al [33], 2019	Physiological data: RR	No	VRE: 5 min; FES: not reported
Tabor et al [8], 2020	Physiological data: RR, inhalation duration, and exhalation duration	No	VRE: not reported; FES: not reported
Tao et al [46], 2020	Quantitative data: Questionnaire for VR experience and Just Noticeable Difference Physiological data: Exhalation detection	Yes	VRE: 5 min; FES: not reported
Tatzgern et al [48], 2022	Quantitative data: Igroup presence questionnaire, Borg CR10 scale, Paas rating scale, Single Ease Question, and VR questionnaire Physiological data: Inhalation and exhalation	No	VRE: not reported; FES: 75 min
Tinga et al [29], 2018	Quantitative data: VAS for calmness Physiological data: RR, HR, HRV, and electroencephalography data	No	VRE: 6 min; FES: not reported
Tu et al [6], 2020	Quantitative data: Questionnaire for training effectiveness and user experience Physiological data: Breathing duration, movement of the chest, HR, and HRV	Yes	VRE: not reported; FES: 45 min
Zafar et al [30], 2018	Physiological data: RR	No	VRE: 6 min; FES: not reported

^a^GAD-7: Generalized Anxiety Disorder-7.

^b^RR: respiratory rate.

^c^HR: heart rate.

^d^VRE: virtual reality exposure.

^e^FES: full experimental session.

^f^VAS: visual analog scale.

^g^STAI: State-Trait Anxiety Inventory.

^h^VR: virtual reality.

^i^IMI: Intrinsic Motivation Inventory.

^j^HRV: heart rate variability.

^k^PSS-10: 10-item Perceived Stress Scale.

^l^HADS: Hospital Anxiety and Depression Scale.

To assess the level of anxiety, the following protocols were used: State-Trait Anxiety Inventory (STAI; 3/32 studies) [25,40,44], Generalized Anxiety Disorder-7 (GAD-7; 2/32 studies) [27,49], Hospital Anxiety and Depression Scale (HADS; 2/32 studies) [42,50], Perceived Stress Scale (PSS; 2/32 studies) [28,39], and Edmonton symptom rating scale (1/32 studies) [50]. The STAI is a 4-point Likert scale form, which contains 40 questions measuring pressure, worry, and anxiety [55,56]. GAD-7 is a 7-question screener that assesses participants’ psychological well-being status [27,57]. The HADS is a 14-item scale that scores 0 to 3 for each item. The first 7 items relate to anxiety, and the remaining 7 relate to depression. A higher score is associated with greater anxiety and symptoms of depression [42]. The PSS is a 14-item scale with 7 positive and 7 negative items rated on a 5-point Likert scale [58]. The Edmonton symptom rating scale is a questionnaire used to rate the intensity of 9 common symptoms experienced by cancer patients, including pain, tiredness, nausea, depression, anxiety, drowsiness, appetite, well-being, and shortness of breath [59]. Moreover, the Cognitive Interference Questionnaire (CIQ) was used to measure the performance, task-oriented worries, and off-task thoughts of participants through a 22-item questionnaire based on a 5-point scale [5,60]. The Mini-Mental State Examination (MMSE; 2/32 studies) is a set of 11 questions to check if participants have cognitive impairments such as problems with thinking, communication, understanding, and memory [61]. Lastly, the visual analog scale (VAS; 2/32 studies) [5,25] was used to evaluate participants’ calmness and relaxation self-efficacy. The VAS is usually used for measuring pain on a 10-point Likert scale, but since the scale is easy to reflect, studies also use it to measure other emotional reflections as well [62].

Some studies (9/32) examined the VR experience [6,10,31,33,38,42,44-46] through self-reported questionnaires. The questionnaires included questions related to the participants’ positive and negative emotions, game flow, engagement, ability, capacity, pressure that the said user was experiencing, and challenges that the user perceived [62,63]. Two studies (2/32) also evaluated the motivation of the participants, using a multidimensional self-reported 7-point scale with a Likert-type format called the Intrinsic Motivation Inventory (IMI) [40,44]. The IMI consists of 7 subscales that measure participants’ experiences related to a target activity, such as interest/enjoyment, perceived competence, effort, value/usefulness, pressure/tension, perceived choice, and relatedness [64]. The Just Noticeable Difference (JND) was used in a study (1/32) to stimulate the perception level of the user on system latency [46]. Another study (1/32) used the Igroup Presence Questionnaire (IPQ) to assess the sense of presence in different VEs [48]. Lastly, an instrument named the State Mindfulness Scale (SMS; 1/32) [25] was designed for mindfulness assessment with 21 questions self-reported on a 7-point Likert scale. In particular, the SMS counts the level of present-moment attention to and awareness of activity (mindful or mental) [65].

#### Biosignals and Physiological Data

Biosignals are physical signals that describe the state of human living. A wide variety of biosignals are regularly used in hospitals and in home monitoring. The most well-known include electrocardiography (ECG), electroencephalography (EEG), and photoplethysmography (PPG). Alternatively, physiological data consist of heart rate (HR), blood pressure, RR, etc.

The studies analyzed the above signals and physiological data in detail. In particular, several studies extracted ECG signals to collect HR data to calculate the average beats per minute [10,25,28,31,37,38,49,50] and HRV to measure the specific changes in time between heart beats [6,29,31,44]. Moreover, a few studies preferred the use of the PPG signal to collect HR [27], blood pressure [50], and oxygen saturation [28,49,50]. Respiration signals were collected by most of the reviewed studies, and they assessed data like lung function [5,36,42], lung volume [8,28], expansion [6,40], and breathing force [48]. Further, respiration signals were used to calculate physiological data, such as RR [8,27,28,30,32,33,35,38-40,45,49,50], and exhalation and inhalation durations [8,39], while accelerometer data were used to identify participants’ chest movements [40]. Moreover, EEG data were collected by researchers to reflect participants’ brain activity [29,38]. Lastly, electrodermal signals were used by a few studies to record the electric characteristics of the skin and allow researchers to assess participants’ stress levels [37,38].

### Apparatus

#### VR Technology

VR allows its users to have full control over the environment they are exposed to. VR technology can provide acoustic, visual, tactile, or olfactory interactions between the user and the system. Based on the above abilities, VR systems can be categorized as nonimmersive, semi-immersive, and fully immersive [66]. Our review found that 21 of the 32 evaluated studies [6,10,25-29,31-33,36,37,39,41,43,45-49] used fully immersive equipment, where the participant’s vision is fully enveloped with a head-mounted display (HMD) system and the interactions with the system are based on natural gesture recognition processes. However, 5 of the 32 studies used nonimmersive VR equipment, such as a smartphone [29,44], tablet [5], or laptop [8,34], and these 3D graphical systems allowed users to navigate VEs. Lastly, 6 of the 32 studies did not report the type of VR equipment used [7,8,24,33,39,40].

#### Biofeedback Equipment

**Figure 2:**
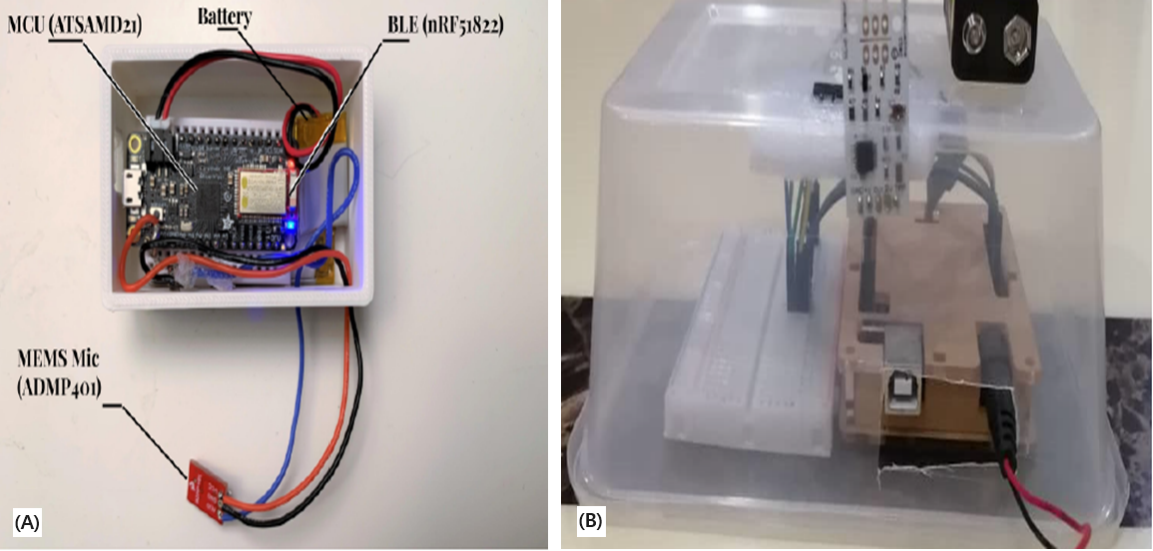
Images of assembled input devices for breathing. (A) The device used a microphone to detect exhalation, which was mapped to the virtual reality (VR) game. (B) The device mapped breathing techniques to the VR game.

All the reviewed studies used biosignal responses to assess the accuracy of the delivered solutions. In particular, the reviewed biosignals were measurements of the physiological changes in respiration, PPG, ECG, EEG, and electrodermal activity. Most of the studies (27/32) used already existing systems to record biosignals [5-8,10,24-34,36-41,44,45,47,49,50]. The search results for biofeedback equipment are presented in Table 4. Impressively, only 4 of the 32 studies developed their own systems [35,43,46,48]. Heng et al [43] developed a breathing input sensor consisting of Arduino Uno, a Rev C. Wind Sensor chip, and an ESP8266 ESP-01S Wi-Fi module. The Arduino Uno board acts as the main processor for the breathing input sensor and the power supplier for the whole system. The wind sensor chip picks up the wind signal from human breath and translates it into raw reading data. After that, the Arduino Uno board translates the raw reading data into wind speed. Finally, ESP-01S communicates with the system software through a WebSocket protocol. Moreover, Hu et al [35] used the same Arduino Uno board as mentioned above but with a different sensor. A gas pressure sensor (XGZP6857A) was applied for respiratory measurements. Tao et al [46] established their own system hardware based on an Adafruit Feather M0 Bluefruit LE board. This board has 2 main features: processing of audio signals with an ATSAMD21G18 ARM Cortex M0 processor and Bluetooth Low Energy communication with an nRF51822 chipset. A MEMS wired microphone was used for the board analog input to read the signal (Figure 2). The system could take breath as input for instrument playing in the VR game. The microphone detected exhalation, which was mapped to the instrument audio in the VR game.

**Table 4:** Virtual reality breathing studies: biofeedback sensors and interactive devices.

Study	Biofeedback sensors	VR^a^ apparatus
Abushakra et al [7], 2014	Unspecified microphone and smartphone	Unspecified apparatus
Betka et al [49], 2022	Go Direct chest strap [67]	Zeiss VR ONEPLUS [68]
Blum et al [25], 2019	Polar H7 chest strap [69]	Oculus Rift CV1 [70]
Blum et al [10], 2020	Polar H10 chest strap [71]	Oculus Rift CV1 [70]
Brammer et al [41], 2021	Unspecified chest strap	Unspecified apparatus
Charoensook et al [31], 2019	Zephyr BioHarness Physiology Monitoring System [72]	HTC Vive [73]
van Delden et al [5], 2020	Air Next (NuvoAir) spirometer [74]	Unspecified apparatus
Feinberg et al [47], 2022	Not reported	Oculus Quest [75]
Gummidela et al [45], 2022	Zephyr BioHarness chest strap [72]	Nexus 6P smartphone [76]
Heng et al [43], 2020	Arduino Uno [77], Rev C. Wind Sensor chip ESP8266 [78], and ESP-01S Wi-Fi module [79]	Unspecified apparatus
Hu et al [35], 2021	Arduino Uno [77], gas pressure sensor (XGZP6857A) [80], and voltage conversion module [81]	Unspecified apparatus
Jung et al [27], 2020	Nonin 3150 probe [82]	VR headset by Pico Interactive Goblin [83]
Kluge et al [36], 2021	EquiVital biosensor [84]	Oculus Rift [85]
Ladakis et al [37], 2021	Scosche Rhythm+ [86] and Moodmetric Ring [87]	Oculus Go [88]
Mevlevioğlu et al [38], 2021	Shimmer device [89] and MyndPlay BrainBand [90]	HTC Vive [73], GeForce GTX Titan X [91] graphics card, and Intel i7-5820k processor [92]
Michela et al [44], 2022	Inductance plethysmography (RIP) belt Plux S.A [93] and Polar H10 chest strap [71]	HTC Vive [73]
Patibanda et al [34], 2017	Breathing+ system sensor [94]	Unspecified apparatus
Prpa et al [26], 2018	Two Thought Technology respiration sensors [95]	Oculus Rift SDK2 [85]
Quintero et al [32], 2019	Samsung smartwatch Gear Sport [96]	Samsung Galaxy S9 [97] and Samsung Gear VR [98]
Rockstroh et al [39], 2021	VR controllers [99]	Oculus Quest VR [75]
Rodrigues et al [50], 2022	Unspecified oximeter and sphygmomanometer equipment	Oculus Realidade Virtual 3D Gamer Warrior JS080 [100]
van Rooij et al [40], 2016	Unspecified stretch sensor	Unspecified apparatus
Rutkowski et al [42], 2021	Unspecified sensor	VR TierOne device [101]
Shih et al [24], 2019	Mindmedia’s NeXus respiration sensor [102]	Unspecified apparatus
Soyka et al [28], 2016	g.RESPsensor Piezo-electric crystal respiration effort sensor [103]	Oculus Rift DK1 [85]
Desnoyers-Stewart et al [33], 2019	Biosignalplux breathing sensor [93]	HTC Vive [73] and a projection (6.5×3.66 m)
Tabor et al [8], 2020	Blue Yeti microphone [104]	Unspecified apparatus
Tao et al [46], 2020	Adafruit Feather M0 Bluefruit LE [80], ATSAMD21G18 ARM Cortex M0 processor [105], and MEMS microphone [106]	Oculus VR [75]
Tatzgern et al [48], 2022	Sensirion SFM3300 mass flow sensor [107], 3M 6000 series respirator mask [108], and Motor SG-90 servomotor [109]	Oculus Quest 2 head-mounted display [75]
Tinga et al [29], 2018	Respiratory effort transducer SS5LB [110] and BIOPAC System Inc wireless B-Alert X10 system (ABM) [111]	Oculus Rift DK2 [85]
Tu et al [6], 2020	Empatica E4 emp [112] and Hexoskin t-shirt [113]	Google cardboard GGC [114]
Zafar et al [30], 2018	Zephyr BioHarness 3.0 chest strap [71]	LG Nexus 4 smartphone [115]

^a^VR: virtual reality.

**Figure 3:**
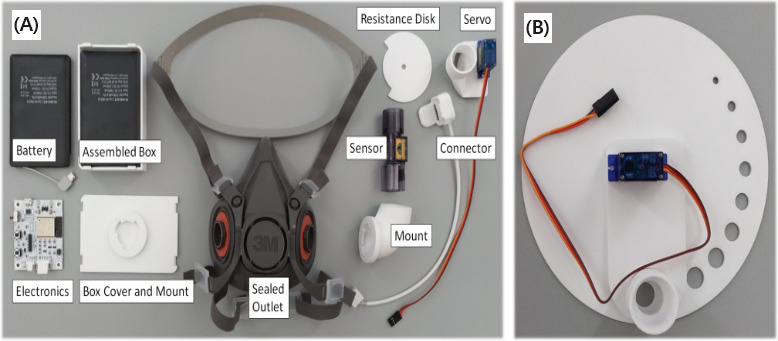
Developed system. (A) The first iteration used a common medical oxygen mask, which did not seal the airflow paths sufficiently to be able to experience breathing resistance. Thus, a safety respirator mask was used. (B) Early design of the disk controlling resistance.

Tatzgern et al [48] created the AirRes mask to measure participants’ breathing according to VE interactions (Figure 3). The system depends on a Sensirion SFM3300 mass flow sensor 2. This sensor allows the assessment of a participant’s airflow with high-precision measurements from both directions (exhalation and inhalation). A protective gear 3M mask frame was used, and the custom-made equipment (electronic circuit) and breathing sensor were added on the frame. Moreover, a disk was applied to the mask to change the amount of air. A Servo Motor SG-90 device was used to rotate and control this disk. A custom circuit board was connected to the airflow sensor and servomotor. The above custom-made system controlled resistance wirelessly in the VR game through an ESP32 Bluetooth module.

### VEs and Interventions

As mentioned above, one of the most important advantages of VR is its ability to provide the feeling of being immersed in a simulated environment [66]. Even though the participants of VR technology acknowledge and recognize that the environment provided by VR is not real, they act as they would in a real environment. The reviewed studies focused on specific types of VEs for mental and anxiety rehabilitation and pulmonary rehabilitation. The study results for VEs are presented in Table 5.

**Table 5:** Virtual reality breathing studies: virtual environments.

Study	Virtual environments
Abushakra et al [7], 2014	Tissue layers and cells are presented to the participant. Through different types of breathing, the participant diminishes the cancerous lung cells.
Betka et al [49], 2022	A room with a matched-gender virtual body lying on a couch. The virtual body is illuminated synchronously or asynchronously according to the patient’s chest movements.
Blum et al [25], 2019	The natural environment of a beach scenery at sunset with palms, rocks, several light sources, and a campfire.
Blum et al [10], 2020	Natural environment with a landscape having hills, flowers, parts of trees, and rocks that change their color according to breathing.
Brammer et al [41], 2021	The trainees must shoot hostile zombies while they leave the benign zombies unharmed.
Charoensook et al [31], 2019	There were 4 games: (1) Beat Saber, (2) Space Pirate Trainer, (3) Gorn, and (4) Final Approach.
van Delden et al [5], 2020	There were 3 games: (1) Popping balloons, (2) Car, and (3) Diving.
Feinberg et al [47], 2022	A dedicated space for meditation with peers and a virtual instructor with an hourglass to track time. There is a bonsai tree that grows to signify progress. The environment changes every few sessions, including weather (sunset or rainstorm).
Gummidela et al [45], 2022	A square arena in which a ball bounces with a randomly initialized direction and location.
Heng et al [43], 2020	There were 8 mini-games: (1) Bubble Gum, (2) Candle Blower, (3) Windmill, (4) Pest Control, (5) Wind Arrow, (6) Table Cleaner, (7) Winter Window, and (8) Steak Gourmet.
Hu et al [35], 2021	There were 2 games: (1) pond scene and (2) ocean scene.
Jung et al [27], 2020	Not reported
Kluge et al [36], 2021	There were 8 discrete modules: (1) emotions, thoughts, and actions; (2) controlled breathing; (3) progressive muscle relaxation; (4) grounding; (5) values and realities; (6) stress reappraisal; (7) managing thoughts/cognitive defusion; and (8) acceptance and avoidance.
Ladakis et al [37], 2021	Fantasy Forest Environment: the participant walks in nature and relaxation sceneries that facilitate recovery from job stress.
Mevlevioğlu et al [38], 2021	There were 2 scenes: (1) nature scene with trees, grass, and flowers moving based on the user’s breathing; and (2) elevator scene with a glass elevator outside of a large building in a city, with a height of 6 levels.
Michela et al [44], 2022	A parking garage with friendly and hostile human targets to shoot or not.
Patibanda et al [34], 2017	Life Tree: a tree starts growing through inhalation and exhalation.
Prpa et al [26], 2018	Pulse Breath Water: ocean waves that change their movement according to breathing pace.
Quintero et al [32], 2019	Calm Place: climate sequence that goes from dusk to noon with the appearance of a blue object in the middle of the virtual scene to guide the breathing exercise.
Rockstroh et al [39], 2021	Two types of virtual environments of nature, with elements such as trees, grass, flowers, and rocks.
Rodrigues et al [50], 2022	A relaxed environment.
van Rooij et al [40], 2016	An underwater world in which children can move around freely and explore at their leisure.
Rutkowski et al [42], 2021	Virtual therapeutic garden.
Shih et al [24], 2019	A sailing boat moving backward and forward with the participant’s breathing.
Soyka et al [28], 2016	A jellyfish moving up and down in an underwater environment.
Desnoyers-Stewart et al [33], 2019	An underwater world with 2 jellyfish and a growing glass sponge.
Tabor et al [8], 2020	There were 2 games: (1) Bubble Float and (2) Bubble Paint.
Tao et al [46], 2020	A music studio scene, where the participant was asked to practice a virtual reality harmonica instrument.
Tatzgern et al [48], 2022	There were 6 scenarios: (1) blowing all candles on a cake, (2) blowing projectiles through a blow tube, (3) shooting a toy gun, (4) blowing ships, (5) inflating balloons, and (6) playing the harmonica.
Tinga et al [29], 2018	A white cloud moving toward and away in the direction of the participant’s mouth.
Tu et al [6], 2020	There were 2 games: (1) Balloon, where the participant could control the movement of a balloon through respiration and (2) Pilot, where the participant’s breathing could control a flight’s course.
Zafar et al [30], 2018	There were 3 video games: (1) Chill Out, (2) Dodging Stress, and (3) Pacman Zen.

### Types of VEs for Mental and Anxiety Rehabilitation

**Figure 4:**
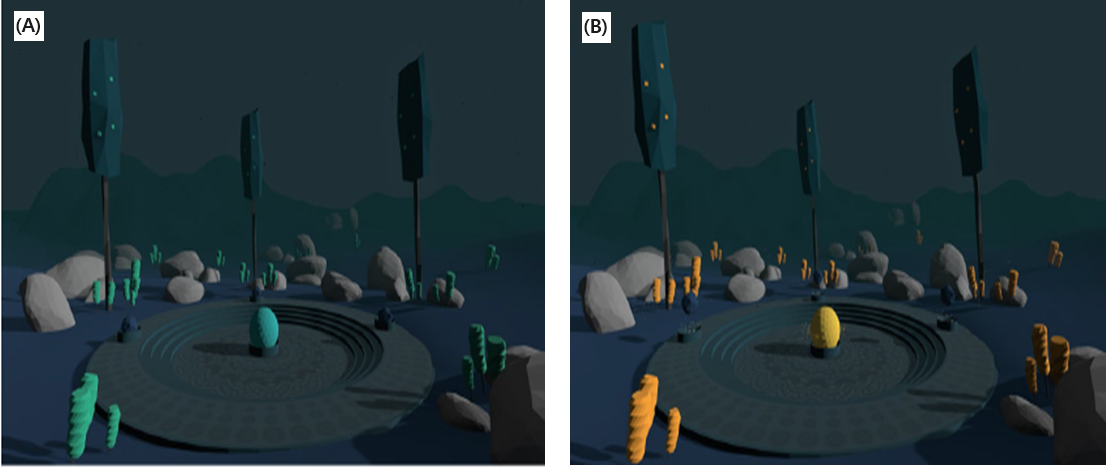
Screenshot of the beach virtual environment in its default state (A) and while exhaling (B).

Numerous studies (12/32) used nature scenes, such as scenes of beaches, forests, oceans, and mountains [10,25,26,28,29,33,34,36-40]. Below, we present 4 of the most impressive natural VEs. The first environment was a beach scenery at sunset with several dynamic parameters like lights and clouds, which shifted according to breathing to provide feedback to the participant (Figure 4) [25]. If the participant’s breathing was below the threshold, which was preset by the system, the breathing pace was considered to be correct. As a result of correct breathing, the sky turned clear, and the participant could enjoy a star-spangled sky. A campfire was also included in the said scenario as a dynamic object to make the participant feel more relaxed and to provide the participant with an indication of whether breathing was at the appropriate pace.

**Figure 5:**
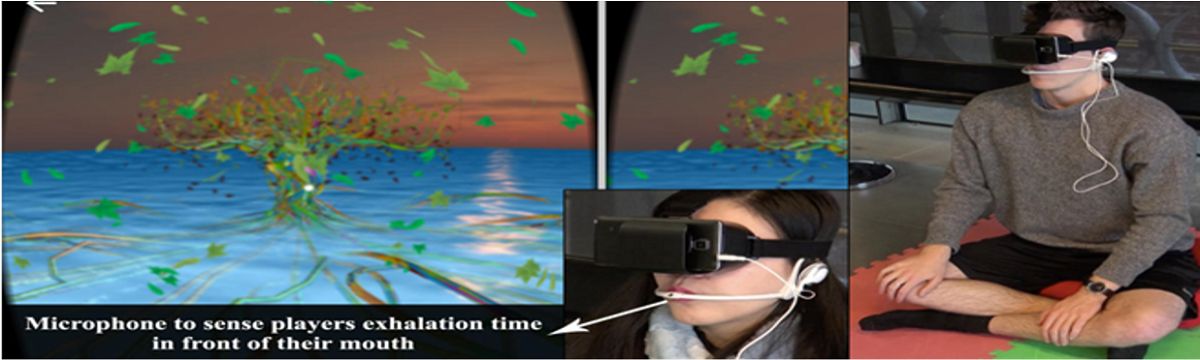
A participant playing the tree game while wearing a breathing headset and a virtual reality head-mounted display.

The second environment was a dynamic scenario with a tree submerged in the middle of water (Figure 5) [34]. The goal of this intervention was to help the participant to practice pursed-lip breathing. The participant was told to wear an unidentified HMD and sit in a comfortable position with the legs crossed. Before the start of the intervention, the participant was advised to go through a breathing exercise introduction. After that, the participant had to follow rhythmic breathing by inhaling and exhaling until the tree started to expand on inhalation and contract on exhalation. If the participant continued to breathe rhythmically, the tree started to bloom. If the breathing of the participant was nonrhythmic, the view of the participant started to blur in the monitor until the right rhythm was found. Leaves were also used to provide feedback to the participant on the breathing rhythm. More colorful leaves indicated that the participant was following the correct breathing rhythm.

**Figure 6:**
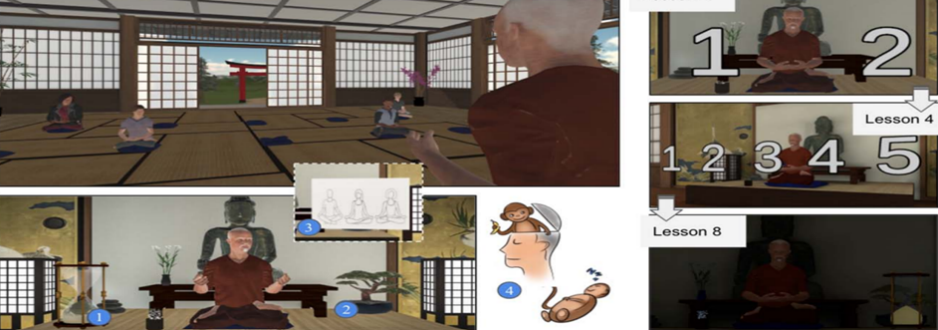
ZenVR learning environment that includes a virtual teacher, an hourglass, a bonsai tree, and numbers to count inhalation and exhalation.

The third environment was the ZenVR environment, which was a dedicated VE that included an open room in a mountain environment with plants, where the participant could train on different meditation techniques (Figure 6) [47]. The VE contained several objects related to meditation like meditation cushions and candles. Several dynamic parameters, such as the weather and a bonsai tree, changed according to the level of the breath training. During the duration of the training, the bonsai tree grew to indicate the participant’s progress. An hourglass was present as a marker of time for the participant and to indicate that the program continued during the silent meditation. Additionally, numbers appeared with different sizes to illustrate the breathing exercises.

**Figure 7:**
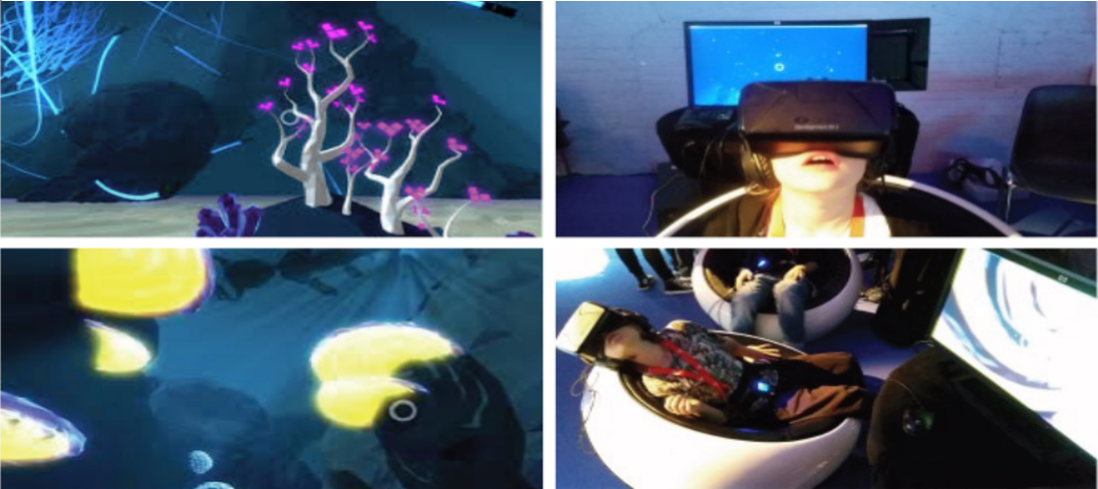
Underwater environment. Screenshots showing the virtual underwater world (left), and pictures of children playing (right).

The last environment was an underwater experience for diaphragmatic breathing practiced with children with anxiety issues (Figure 7) [40]. The participant was instantaneously informed of the state of breathing by a dynamic circle in the VE that expanded according to breathing. The system applied gravity to the participant’s avatar if the lung capacity was more than half. The participant’s breathing pace was able to determine the direction and magnitude of the force. When the participant inhaled, an upward force was applied, and when the participant exhaled, an extra forward force was applied so that the participant was able to dive into the deep ocean. The combination of slow and deep breathing allowed the participant to swim better and have more control in the game. The type of VR equipment was not reported in the study.

**Types of VEs for Pulmonary Rehabilitation**

**Figure 8:**
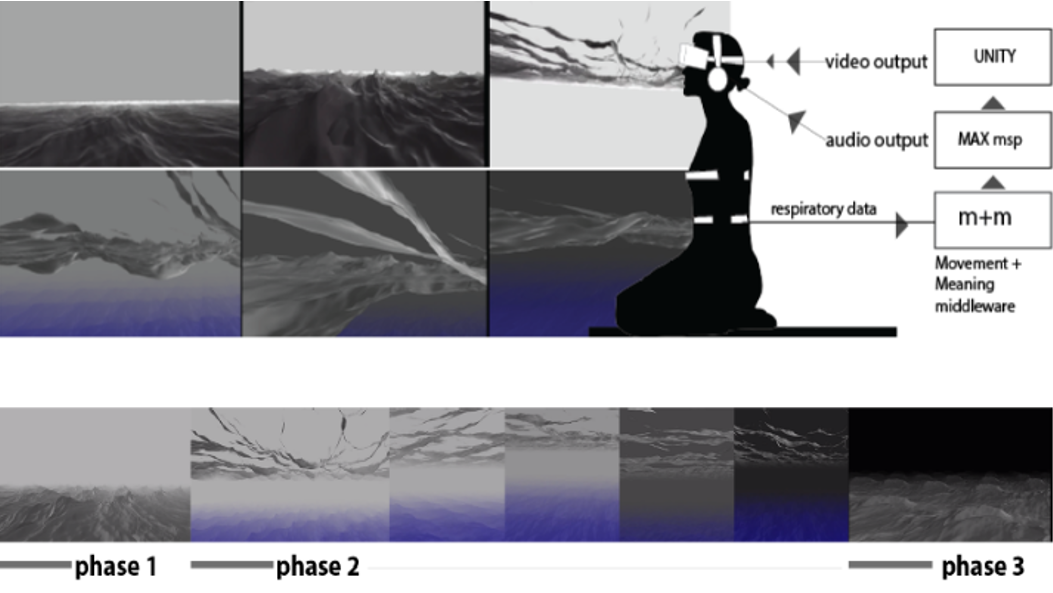
Developed virtual environment. Phase 1 starts with a light grey sky at a stationary participant position above the ocean surface. The participant’s breath activates the water element in the virtual environment in phases 2 and 3.

Several studies (10/32) developed different VEs for breathing exercises to improve respiration [5,6,8,29,31,32,34,41,47,49]. In particular, Prpa et al [26] generated a 3D element of a body of water (an ocean) (Figure 8). This minimal environment displayed the ocean and the sky through a variety of grayscale shades. A continuous breathing pattern allowed the participant to control the ocean environment. It started with a light grey sky and a stationary participant position above the ocean surface. When the participant found the breathing flow, the game continued with movements of ocean waves, which were based on the participant’s breathing pace. The right breathing pattern was mapped to wave movement and musical rhythm. The sky changed from grey to black until the ocean was stationary again, as was seen by the participant at the beginning of the procedure, with the only difference being the color of the sky.

**Figure 9:**
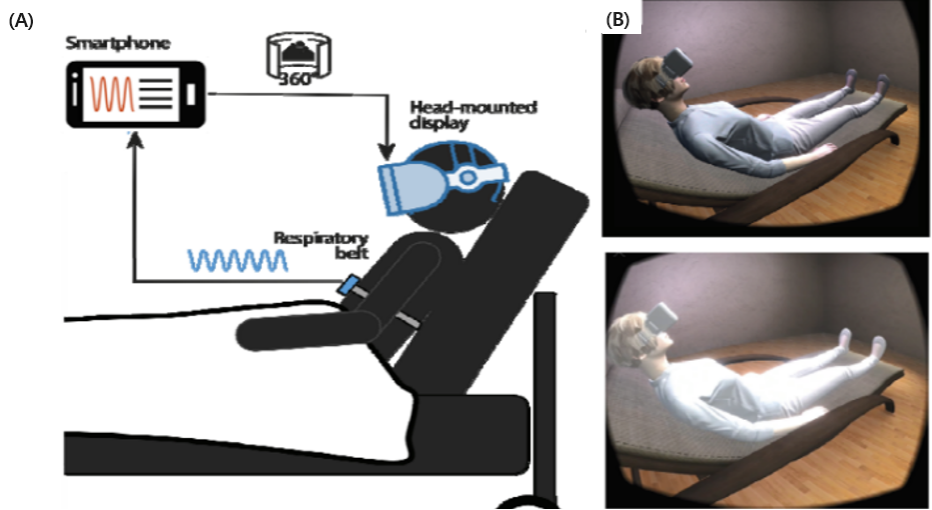
The virtual reality system developed for alleviating dyspnea. (A) Scheme showing the real posture of the patient and the biosignal devices. (B) Representation of the virtual body and how the body’s luminosity changes according to the patient’s chest movements.

Another study developed a VR intervention for alleviating dyspnea in patients recovering from COVID-19 pneumonia (Figure 9) [49]. Specifically, it created a room with a matched gender body lying on a couch. The goal was to illuminate the virtual body synchronously or asynchronously according to the patient’s chest movements.

**Figure 10:**
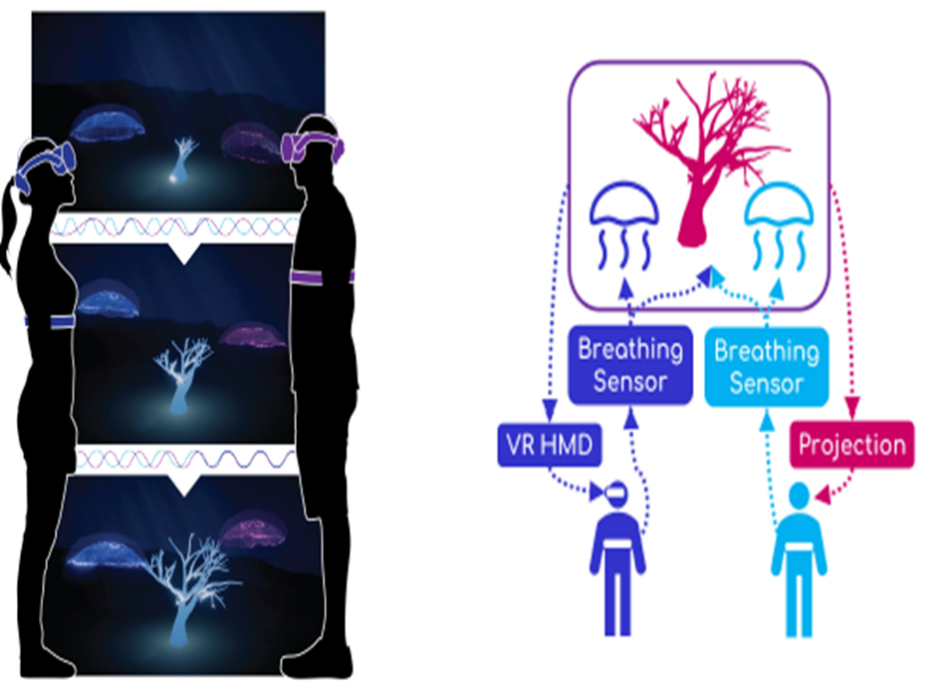
The multi-user virtual reality JeL system. (A) Two jellyfish agents and a growing glass sponge. The jellyfish respond directly to each user’s breathing, while the sponge reflects the synchronization of their breath. HDM: head-mounted display; VR: virtual reality.

A particularly interesting study used multi-user VR to simultaneously immerse two or more participants into an underwater world with jellyfish and a growing glass sponge (Figure 10) [33]. The aim was to synchronize the breathing between the participants, and enhance the breathing awareness, breathing pace, and relation between the participants. In this environment, each participant’s breath was represented by a jellyfish, which moved and glowed in such a way as to provide clear breathing feedback. As the participants synchronized their breathing, the glass sponge was structured to begin to grow and emit light.

**Figure 11:**
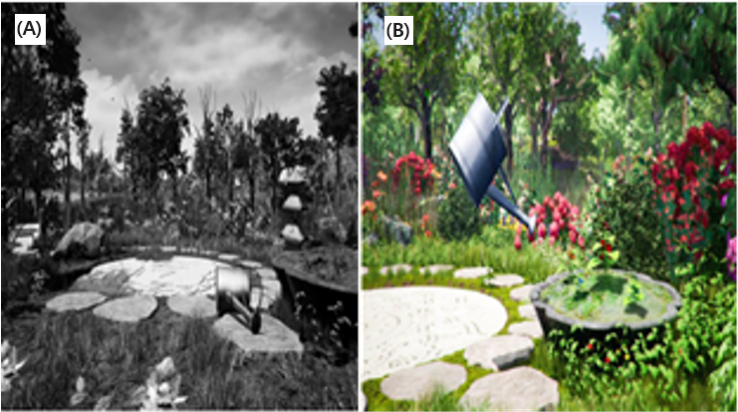
The virtual therapeutic garden game. (A) Initial stage of the game in grayscale. (B) Final stage of the game with the garden full of color.

Interestingly, 2 of the reviewed studies used existing predeveloped systems for pulmonary rehabilitation [42,43]. Rutkowski et al [42] used the virtual therapeutic garden game, released by the European Association of Psychotherapy (Figure 11). Initially, the garden appears grey (untidy and unkept), and the watering pot is on its side. Diaphragmatic exercises with resistance, prolonged exhalation exercises, and chest percussion activate the watering pot to water the garden. With each rehabilitation session and the correct conduct of fitness exercises, the garden becomes increasingly colorful and alive, symbolizing the process of gaining health through the rehabilitation sessions.

**Figure 12:**
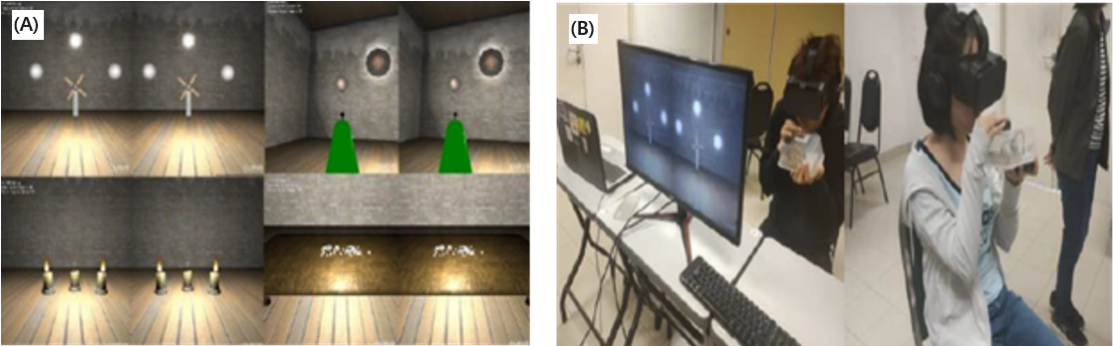
The games in Bubble Tower. (A) Four games are presented. (B) Game users.

Bubble Tower [43] investigated the development of mapping breathing techniques in VR gameplay mechanics (Figure 12). The whole platform offered a set of 8 mini-games, where Bubble Tower was designed to train the participant in the fundamentals of breathing techniques. The mini-games teach the participant basic breathing skills like long and strong breathing, and breathing strength control. These mini-games include stages where the participant is required to pop the bubble with a strong breath, blow candles in a room with a long breath, build up the momentum for a windmill to spin fast with a long breath, etc.

**Figure 13:**
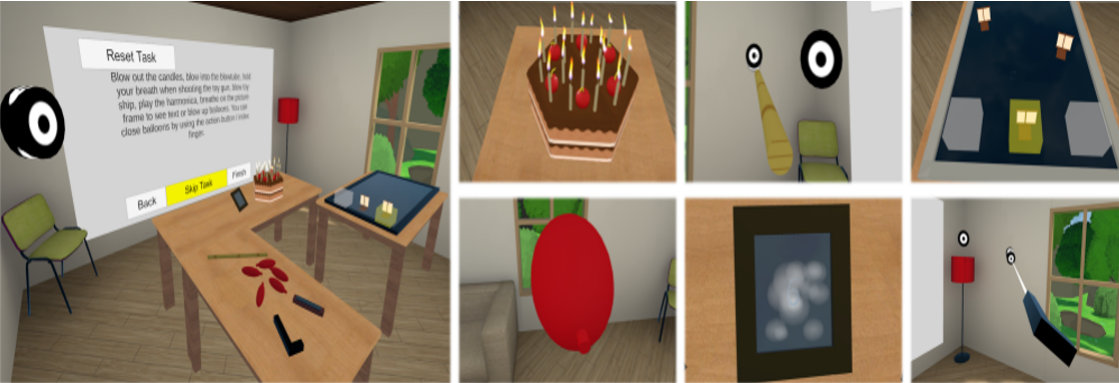
The virtual environment based on the escape room game philosophy. The large image shows the play area of all objects. The other images show some tasks from the game.

An interesting study presented a VE based on the escape room game philosophy (Figure 13) [48]. In the escape room scenario, the participant used different breathing pattern interactions to answer a sequence of tasks to escape from the room. The room had all the objects that the participant needed to escape. During the training, the participant interacted with different individual objects. The first task was to blow out candles on a cake, and then, the participant had to blow bullets through a blow tube toward a target. The third task included the movement of ships based on breathing force. The participant continued with inflating balloons and sorting them by resistance. After that, the participant had to reveal numbers on a mirror with the breath. The last task involved shooting with a toy gun by holding the breath.

## Discussion

### Principal Findings

Based on all the studies that were reviewed (29 studies had a positive label and 3 had a neutral label), it is suggested that VR can be an effective solution for pulmonary rehabilitation among patients with lung cancer, patients with COPD, patients with asthma, and individuals and children who are dealing with mental health–related disorders such as anxiety. Overall, the results indicated that VR can enhance the functional outcomes of pulmonary rehabilitation, increase breathing body awareness, and improve relaxation techniques. In the COVID-19 crisis, evidence showed the need for VR technology adoption. VR pulmonary rehabilitation presents an opportunity for the safe and effective recovery of COVID-19 patients and survivors at home. This technology could be adopted on a large scale to further develop the well-being of individuals during pandemics like COVID-19 and could similarly advance autonomous medical care. In the reviewed studies, most of the VR systems included features of natural environments, like a beach, sky, and forest. Further, it is highly recommended for future studies to incorporate water elements (eg, bubbles) and undersea sceneries (eg, seabed, jellyfish, and sea plants) to enhance relaxation. Some systems involved the use of music and vibrant colors. As technology continues to advance and progress, it is expected that biofeedback in VR systems will have a vital role in the practice of breathing in both the medical setting and the real world. The reviewed studies proved the feasibility of implementing a VR system for pulmonary rehabilitation enhanced with biofeedback. Such a system can be a reliable solution to enhance participant training. An assortment of minimal-cost sensors and biofeedback frameworks can incorporate VR and provide exact and significant information from tasks in gamified biofeedback interventions for breathing. Regarding the adequacy of pulmonary rehabilitation, biofeedback VR innovations must bridge explicit obstacles like equipment assembly, participant population, experiment length, and breathing patterns. The boundless breathing direction in VR frameworks can cause issues in the legitimate decisions of breath-detection equipment. Accordingly, many specialists battle with equipment assembly, equipment incorporation, and its compatibility with VR systems. There have been hindrances in adopting accepted procedures of biofeedback VR in various populations requiring breathing training for different purposes, including overcoming mental health issues and stress pressure, and achieving overall health benefits. The restricted length of examinations can prevent the assessment of the longer-term impacts of pulmonary rehabilitation.

Most of the studies (14/32) examined the effectiveness of breathing through VR based on physiological data [6-8,24,25,29,35,36,43-45,47-49]. Overall, the review suggests that VR is a reliable and feasible solution for pulmonary rehabilitation. Specifically, one of the reviewed studies [8] revealed that VR rehabilitation can be a reliable solution to treat pneumonia. Moreover, it was found that participants who performed pulmonary rehabilitation through VR felt more confident compared to those who performed the training with face-to-face supervision from health care professionals [25]. It was explained that embodied interactions through VR and biofeedback responses made the participants more aware of their inhalation and exhalation rhythm [28]. Additionally, the reviewed studies about pulmonary rehabilitation in COVID-19 patients reported significant improvements in tiredness, shortness and comfort of breath, and vital signs, such as HR, RR, blood pressure, and SpO2 [48,49]. A study comparing patients with COPD undergoing VR pulmonary rehabilitation and those not undergoing this rehabilitation found that VR rehabilitation was associated with high stress release and a sharp reduction in depressive episodes [41]. The positive impact of VR on the enhancement of rehabilitation with different breathing patterns in emotional well-being has been documented by several studies (13/32), with most of these studies being focused on anxiety and stress monitoring [5,8,10,27,28,31,33,34,36,37,39-41,43,49]. The level of anxiety of participants was found to decrease within few minutes after using VR with biofeedback for controlling breathing. In particular, a study comparing induced anxiety between participants who used VR and those who did not use VR reported a reduction in the level of anxiety in those who used VR [39]. In addition, a study documented that apart from the positive effect VR has on anxiety, it can also increase concentration and positively motivate participants [54]. In particular, it was found that participants who performed pulmonary rehabilitation via VR were less reluctant to participate in the training activities. Anxiety levels have also been studied in young populations [39] and COVID-19 patients [49]. VR was suggested to be an effective solution for children at risk for anxiety disorders [39] and was reported to be effective at improving anxiety and increasing the feeling of well-being in patients with COVID-19 [49].

A study reported that participants were able to perform paced breathing techniques without distraction [27]. It suggested the use of vibrant, rich, and multi-dimensional VEs to deliver an effective and enjoyable VR experience. Another study mentioned that water manifestation is a key element to decrease anxiety. More specifically, it was reported that having water features can enhance stress management techniques and significantly expand HRV based on paced breathing [27,54]. Two studies suggested that participants were able to more accurately control the pace of their breathing on adding biofeedback [28,30]. Zafar et al [30] noticed that participants who were exposed to biofeedback systems were able to control their breathing more precisely as opposed to the traditional type of training. Correspondingly, participants of VR biofeedback systems scored higher in their subsequent stress test compared with the pretest. Furthermore, a study that examined the effectiveness of respiratory biofeedback during VR meditation by measuring EEG and ECG signals in a respiratory biofeedback state, control feedback stress state, and control no feedback state, proved that VR meditation is effective for relaxation and breathing exercises. The study findings suggest that if VR is used for meditation, no biofeedback equipment is needed to reduce arousal, providing a more affordable and less intrusive option to apply VR to relaxation exercises [28]. In summary, biofeedback is recommended for the effective deployment of VR systems for pulmonary rehabilitation. The feasibility and findings of the studies are presented in Table 6.

**Table 6:** Virtual reality breathing studies: feasibility and findings.

Study	Feasibility	Findings	Label
Abushakra et al [7], 2014	VR^a^ breathing therapy in real time	85% accuracy	Positive
Betka et al [49], 2022	VR respiratory rehabilitation for COVID-19 patients	Improvement in breathing comfort and enhancement of dyspnea recover.	Positive
Blum et al [25], 2019	VR breath gaming for stress monitoring	Increase in relaxation self-efficacy and reduction in mind wandering.	Positive
Blum et al [10], 2020	VR for breathing exercise	Satisfactory user experience, breath awareness, and greater focus on slow diaphragmatic breathing.	Positive
Brammer et al [41], 2021	VR for breathing-based stress training for police officers	Illustrated the feasibility of stress exposure biofeedback with examples of training in police officers.	Positive
Charoensook et al [31], 2019	VR system for physical fitness improvement	Significant difference in the average heart rate between traditional systems and the VR system.	Neutral
van Delden et al [5], 2020	VR for lung function tracking in children with asthma	100% of the estimated volume goal (full exhalation).	Positive
Feinberg et al [47], 2022	VR for breathing training through meditation	Quantitative and qualitative indicators showed an increase in meditation ability after completing the sessions.	Positive
Heng et al [43], 2020	VR for pulmonary rehabilitation	Minor technical issue with the sensor device.	Neutral
Hu et al [35], 2021	VR for pulmonary rehabilitation in children	Increase in motivation among children and improvement in their adherence to breathing exercises.	Positive
Gummidela et al [45], 2022	VR for relaxation training	Nongame interventions were better at promoting moment relaxation. Game-based interventions were more successful at promoting deep breathing during stressful tasks.	Positive
Jung et al [27], 2020	VR for COPD^b^ rehabilitation	Improvements in the physical ability and psychological well-being of participants.	Positive
Kluge et al [36], 2021	VR for stress management in defense force groups	VR-based apps can develop stress management skills in a workplace setting.	Positive
Ladakis et al [37], 2021	VR for stress reduction in a work environment	VR can be a simple and useful tool for the immediate decrease of stress in various real-life environments.	Positive
Mevlevioğlu et al [38], 2021	VR for height exposure (acrophobia)	A correlation between arousal and virtual height showed that the developed VR experience is capable of producing the wanted effect.	Positive
Michela et al [44], 2022	VR for stress management in police officers	Improvement in breathing control, with a positive effect on breathing-induced low-frequency HRV^c^.	Positive
Patibanda et al [34], 2017	VR for breath gaming	Relaxation of mood among participants.	Positive
Prpa et al [26], 2018	VR for breathing awareness	Awareness of breathing while playing on the VR system.	Positive
Quintero et al [32], 2019	VR for slow-paced breathing exercises to support mental health	Higher relaxation level of participants during a no biofeedback VR scenario.	Neutral
Rockstroh et al [39], 2021	VR for fostering diaphragmatic breathing	VR-based breathing training increased perceived breath awareness, improved diaphragmatic breathing, increased relaxation, decreased perceived stress, and reduced symptoms of burnout.	Positive
Rodrigues et al [50], 2022	VR for controlling dyspnea and pain symptoms in hospitalized patients with COVID-19	Tiredness, shortness of breath, and anxiety decreased, and the feeling of well-being increased.	Positive
van Rooij et al [40], 2016	VR for breathing therapy to reduce anxiety in children	Decrease in self-reported anxiety.	Positive
Rutkowski et al [42], 2021	VR for pulmonary rehabilitation	Reduction in stress and emotional tension between prerehabilitation and postrehabilitation.	Positive
Shih et al [24], 2019	VR for breath gaming to strengthen cardiac functioning	75.5% accuracy in breathing phase detection.	Positive
Soyka et al [28], 2016	VR for home breathing therapy for stress	Improvement in stress monitoring techniques and increase in HRV.	Positive
Desnoyers-Stewart et al [33], 2019	VR for breath gaming to mediate physiological synchrony for social connection	Positive outcomes suggest that the system is functional.	Positive
Tabor et al [8], 2020	VR for respiratory pneumonia rehabilitation	The system makes use of state-of-the-art breath-sensing techniques without specialized sensing hardware.	Positive
Tao et al [46], 2020	VR for playing a music instrument with breath	The latency perception threshold is higher for inexperienced participants than experienced participants.	Positive
Tatzgern et al [48], 2022	VR for interacting with different scenarios with breathing patterns	The system can enhance the training experience and improve breathing awareness.	Positive
Tinga et al [29], 2018	VR for breath gaming for meditation	Reduction in anxiety and stress.	Positive
Tu et al [6], 2020	VR for home breath gaming	Errors lower than 0.61 s and 15 ms, and improvement in training effectiveness and experience.	Positive
Zafar et al [30], 2018	Video biofeedback system to teach breathing control	Biofeedback led to a better attentional-cognitive performance and helped participants to learn breathing control.	Positive

^a^VR: virtual reality.

^b^COPD: chronic obstructive pulmonary disease.

^c^HRV: heart rate variability.

### Limitations and Future Work

Even though the effectiveness of VR for pulmonary rehabilitation is well documented, several limitations have been reported in the reviewed studies. First, some of the reviewed studies measured the relaxing effect of VR through the use of psychophysiological responses, such as HR, and self-reported questionnaires. However, some of the studies suggested the need for additional instruments that explicitly assess different aspects of affect and mood [10,41]. For example, a study suggested the collection of 2 different biosignals (PPG and ECG) for HR accuracy [10]. Moreover, the study mentioned that a participant’s respiration has limited validation and recommended the use of an additional belt sensor [10]. It was suggested to measure stress levels using not only traditional reports, such as self-reports and the Stroop task, but also cortisol levels [41]. Future studies should consider triangulating the physiological data with interviews and other qualitative data to provide a holistic assessment of the impact of biofeedback systems [29,44]. Future studies can also investigate the factors that can enhance VR pulmonary rehabilitation to support existing relaxation and destress techniques, and this should be compared to traditional practices [27].

Second, the reviewed studies highlighted the limit of breathing guidance in VR systems, which can provoke complications and barriers in the correct choice of breathing sensing hardware [45,47]. As a result, many researchers struggled with aspects of the hardware apparatus, like the weight of the hardware on the user’s head in addition to the VR HMD [47], as well as with hardware integration and its compatibility with VR systems [45]. In addition, most hardware equipment involved high-end solutions, and the cost for the equipment in most of the studies was between €300 and €1200 (US $322 and US $1289, respectively) [5,6,10,27,29-33,36-39,45]. Three studies [34,46,47] developed their own affordable equipment, for which the cost was approximately €70 (US $75). Future studies should provide clear guidelines for the effective apparatus as well as the cost of the system. As mentioned previously [66], moving to low-cost and accessible solutions will decrease the need for technical support. This suggests that participants will be able to have their own personalized devices, which could lead to an increased quality of life.

It was further suggested for future studies to build systems that include machine learning algorithms to empower participant rehabilitation. These kinds of systems can offer the advantage of higher relaxation levels. For this, it is recommended for model algorithms to automatically classify breathing states, compared with other sensors, and analyze different visual cues in VEs. Moreover, it was stated that machine learning methods should adapt logic modules to provide automatic adaptations in VEs [31]. It is worth mentioning that Fast Fourier Transformation implementation presents a limitation compared with other methods of low-frequency detection, although it is fully functional. For future studies, it is therefore suggested to implement a wavelet transform method, which can provide higher resolution at low frequencies without requiring a larger window size [33].

To improve the systems even further, future studies should examine the effectiveness of biofeedback compared with proper control conditions in different groups of participants under different types of circumstances to determine exactly when and why biofeedback might not be preferable. For example, the effects of biofeedback in children, as examined in the study by van Rooij et al [40], could differ from the effects of biofeedback in adults, as examined by Tinga et al [29]. In the study by Tinga et al [29], the reduction in arousal (on all outcome measures combined and HR specifically) was the largest in the control feedback placebo condition, indicating that respiratory biofeedback had no additional value in reducing arousal and was even less effective than the control feedback placebo. The above finding indicates no preference for respiratory biofeedback compared with control feedback placebo in lowering pain levels in participants with chronic back pain.

Third, a study suggested the extension of VR exposure time, since it was found that this might allow participants to develop their own strategies for producing respiration patterns [31]. Expectedly, most studies suggested that a large sample is required to verify the trend of average HRV and other bioindicators [30,43], as well as to address difficulties in VR design [8,45]. An enhanced sample size could have a positive impact on VR design since a wider set of participants can express their interests [48,49]. Finally, it has been suggested for future studies to extend experiments to multi-session investigations to examine the longer-term effects of pulmonary rehabilitation among participants [25,30,33,49].

### Conclusion

The future directions of biofeedback VR technologies hold huge potential for significant advancements in the pulmonary field, ushering in a new era of personalized and adaptive experiences, enhanced sensor technologies, integration with artificial intelligence and machine learning, gamification and immersive exercises with integration into telehealth, and remote monitoring.

One of the most exciting prospects of VR is the ability to deliver personalized and adaptive experiences through biofeedback VR technologies. VR applications can dynamically tailor experiences to patients based on their specific needs. Real-time integration of user responses and physiological data enables these applications to optimize the effectiveness of biofeedback interventions, ensuring that patients receive the most relevant and impactful feedback. Advancements in sensor technologies are another crucial area of development. Wearable sensors, such as biometric devices, provide real-time data on BR, HR, skin conductance, muscle tension, etc. Continued improvements in sensor miniaturization, wireless connectivity, and comfort will enhance the usability and reliability of biofeedback VR technologies, making them more accessible and user friendly.

The integration of artificial intelligence and machine learning presents exciting possibilities for biofeedback VR technologies. These algorithms can analyze massive amounts of biofeedback data, identify patterns, and provide personalized recommendations for stress reduction, relaxation breathing techniques, or breath performance enhancement. Machine learning can also help in monitoring progress, evaluating outcomes, and optimizing the effectiveness of biofeedback interventions. The cooperation between artificial intelligence, machine learning, and biofeedback VR technologies has the potential to unlock new levels of personalized and evidence-based interventions. Gamification and immersive experiences play crucial roles in engaging users and maximizing the benefits of biofeedback VR technologies. By incorporating game elements and designing interactive VEs, biofeedback VR applications can provide engaging and motivating experiences.

The reviewed studies showed that VR technology can be applied in various areas in the health field, such as stress management, anxiety disorders, pain management, phobia treatment, and rehabilitation. It is necessary to establish evidence-based practices and guidelines for the use of biofeedback VR technologies in health care through collaboration among researchers, health care professionals, and developers. The integration of biofeedback VR technologies in clinical settings will revolutionize the way doctors and researchers approach treatment, improving accessibility, reducing health care costs, and enhancing patient engagement and outcomes.

In the future, innovations in biofeedback VR technologies may also include developments in neurofeedback and brain-computer interfaces with the brain activity of patients. As technology continues to grow and VR progress is investigated, these future directions are poised to shape the field, leading to transformative applications and advancements in wellness, health care, education, etc. The continued exploration and integration of VR technologies and biofeedback technologies have the potential to revolutionize how we understand and enhance human performance, well-being, and quality of life in the future.

## Appendix

Multimedia Appendix 1 PRISMA (Preferred Reporting Items for Systematic Reviews and Meta-Analyses) checklist.

## Abbreviations

COPD: chronic obstructive pulmonary disease
ECG: electrocardiography
EEG: electroencephalography
GAD-7: Generalized Anxiety Disorder-7
HADS: Hospital Anxiety and Depression Scale
HMD: head-mounted display
HR: heart rate
HRV: heart rate variability
IMI: Intrinsic Motivation Inventory
PPG: photoplethysmography
PSS: Perceived Stress Scale
RR: respiratory rate
SMS: State Mindfulness Scale
STAI: State-Trait Anxiety Inventory
VAS: visual analog scale
VE: virtual environment
VR: virtual reality

## Data Availability

The data sets generated and analyzed during this study are available from the corresponding author upon reasonable request.

## References

[1] Elliott WJ, Izzo JL (2006). Device-guided breathing to lower blood pressure: case report and clinical overview. MedGenMed.

[2] Lin I (2018). Effects of a cardiorespiratory synchronization training mobile application on heart rate variability and electroencephalography in healthy adults. Int J Psychophysiol.

[3] Lee PS (1999). Theoretical Bases and Technical Application of Breathing Therapy in Stress Management. J Korean Acad Nurs.

[4] Raab K (2014). Mindfulness, self-compassion, and empathy among health care professionals: a review of the literature. J Health Care Chaplain.

[5] van Delden R, Plass-Oude Bos D, de With AJV, Vogel K, Klaassen R, Zwart N, Faber J, Thio B, van der Kamp M (2020). CHI PLAY '20: Proceedings of the Annual Symposium on Computer-Human Interaction in Play.

[6] Tu L, Hao T, Bi C, Xing G (2020). BreathCoach: A smart in-home breathing training system with bio-feedback via VR game. Smart Health.

[7] Abushakra A, Faezipour M (2014). Augmenting Breath Regulation Using a Mobile Driven Virtual Reality Therapy Framework. IEEE J. Biomed. Health Inform.

[8] Tabor A, Pradantyo R, Sadprasid B, Birk M, Scheme E, Bateman S (2020). Bubble Breather - A Breathing Exercise Game to Support Pneumonia Rehabilitation and Recovery. CHI PLAY '20: Extended Abstracts of the 2020 Annual Symposium on Computer-Human Interaction in Play.

[9] Weerdmeester J, van Rooij M, Harris O, Smit N, Engels R, Granic I (2017). Exploring the Role of Self-efficacy in Biofeedback Video Games. CHI PLAY '17 Extended Abstracts: Extended Abstracts Publication of the Annual Symposium on Computer-Human Interaction in Play.

[10] Blum J, Rockstroh C, Göritz A (2020). Development and Pilot Test of a Virtual Reality Respiratory Biofeedback Approach. Appl Psychophysiol Biofeedback.

[11] Pandita S, Won A (2020). Chapter 7 - Clinical applications of virtual reality in patient-centered care. Technology and Health: Promoting Attitude and Behavior Change.

[12] Chittaro L, Sioni R (2014). Evaluating mobile apps for breathing training: The effectiveness of visualization. Computers in Human Behavior.

[13] WHO Coronavirus (COVID-19) Dashboard. World Health Organization.

[14] Matamala-Gomez M, Bottiroli S, Realdon O, Riva G, Galvagni L, Platz T, Sandrini G, De Icco R, Tassorelli C (2021). Telemedicine and Virtual Reality at Time of COVID-19 Pandemic: An Overview for Future Perspectives in Neurorehabilitation. Front Neurol.

[15] Sampaio M, Haro M, De Sousa B, Melo W, Hoffman HG (2021). Therapists Make the Switch to Telepsychology to Safely Continue Treating Their Patients During the COVID-19 Pandemic. Virtual Reality Telepsychology May Be Next. Front Virtual Real.

[16] Zhang W, Paudel D, Shi R, Liang J, Liu J, Zeng X, Zhou Y, Zhang B (2020). Virtual Reality Exposure Therapy (VRET) for Anxiety Due to Fear of COVID-19 Infection: A Case Series. Neuropsychiatr Dis Treat.

[17] Imperatori C, Dakanalis A, Farina B, Pallavicini F, Colmegna F, Mantovani F, Clerici M (2020). Global Storm of Stress-Related Psychopathological Symptoms: A Brief Overview on the Usefulness of Virtual Reality in Facing the Mental Health Impact of COVID-19. Cyberpsychol Behav Soc Netw.

[18] Jaywant A, Vanderlind WM, Boas SJ, Dickerman AL (2021). Behavioral interventions in acute COVID-19 recovery: A new opportunity for integrated care. Gen Hosp Psychiatry.

[19] da Silva TD, de Oliveira PM, Dionizio JB, de Santana AP, Bahadori S, Dias ED, Ribeiro CM, Gomes RDA, Ferreira M, Ferreira C, Silva DMM, Barnabé V, de Araújo L, Santana HBR, Monteiro CBDM, de Moraes (2021). Comparison Between Conventional Intervention and Non-immersive Virtual Reality in the Rehabilitation of Individuals in an Inpatient Unit for the Treatment of COVID-19: A Study Protocol for a Randomized Controlled Crossover Trial. Front Psychol.

[20] Rutkowski S (2021). Management Challenges in Chronic Obstructive Pulmonary Disease in the COVID-19 Pandemic: Telehealth and Virtual Reality. J Clin Med.

[21] Zampogna E, Paneroni M, Belli S, Aliani M, Gandolfo A, Visca D, Bellanti M, Ambrosino N, Vitacca M (2021). Pulmonary Rehabilitation in Patients Recovering from COVID-19. Respiration.

[22] Gloeckl R, Leitl D, Jarosch I, Schneeberger T, Nell C, Stenzel N, Vogelmeier CF, Kenn K, Koczulla AR (2021). Benefits of pulmonary rehabilitation in COVID-19: a prospective observational cohort study. ERJ Open Res.

[23] Siddiq MAB, Rathore FA, Clegg D, Rasker JJ (2020). Pulmonary Rehabilitation in COVID-19 patients: A scoping review of current practice and its application during the pandemic. Turk J Phys Med Rehabil.

[24] Shih CH, Tomita N, Lukic YX, Reguera Á, Fleisch E, Kowatsch T (2020). Breeze: Smartphone-based Acoustic Real-time Detection of Breathing Phases for a Gamified Biofeedback Breathing Training. Proceedings of the ACM on Interactive, Mobile, Wearable and Ubiquitous Technologies.

[25] Blum J, Rockstroh C, Göritz A (2019). Heart Rate Variability Biofeedback Based on Slow-Paced Breathing With Immersive Virtual Reality Nature Scenery. Front Psychol.

[26] Prpa M, Tatar K, Françoise J, Riecke B, Schiphorst T, Pasquier P (2018). Attending to Breath: Exploring How the Cues in a Virtual Environment Guide the Attention to Breath and Shape the Quality of Experience to Support Mindfulness. DIS '18: Proceedings of the 2018 Designing Interactive Systems Conference.

[27] Jung T, Moorhouse N, Shi X, Amin MF (2020). A Virtual Reality-Supported Intervention for Pulmonary Rehabilitation of Patients With Chronic Obstructive Pulmonary Disease: Mixed Methods Study. J Med Internet Res.

[28] Soyka F, Leyrer M, Smallwood J, Ferguson C, Riecke B, Mohler B (2016). Enhancing stress management techniques using virtual reality. SAP '16: Proceedings of the ACM Symposium on Applied Perception.

[29] Tinga A, Nyklíček I, Jansen M, de Back T, Louwerse M (2019). Respiratory Biofeedback Does Not Facilitate Lowering Arousal in Meditation Through Virtual Reality. Appl Psychophysiol Biofeedback.

[30] Zafar MA, Ahmed B, Rihawi RA, Gutierrez-Osuna R (2020). Gaming Away Stress: Using Biofeedback Games to Learn Paced Breathing. IEEE Trans. Affective Comput.

[31] Charoensook T, Barlow M, Lakshika E (2019). Heart Rate and Breathing Variability for Virtual Reality Game Play.

[32] Quintero L, Papapetrou P, Muñoz JE (2019). Open-Source Physiological Computing Framework using Heart Rate Variability in Mobile Virtual Reality Applications.

[33] Desnoyers-Stewart J, Stepanova E, Pasquier P, Riecke B (2019). JeL: Connecting Through Breath in Virtual Reality. CHI EA '19: Extended Abstracts of the 2019 CHI Conference on Human Factors in Computing Systems.

[34] Patibanda R, Mueller F, Leskovsek M, Duckworth J (2017). Life Tree: Understanding the Design of Breathing Exercise Games. CHI PLAY '17: Proceedings of the Annual Symposium on Computer-Human Interaction in Play.

[35] Hu Y, Zhao Y, Shao Y, Zhu C, Chen J, Shi T, Zhou Z, Ying F, Wang G, Yao C (2021). Bubble Beats: A Breathing Exercise Game Based on Music Rhythm for Children. IDC '21: Proceedings of the 20th Annual ACM Interaction Design and Children Conference.

[36] Kluge M, Maltby S, Walker N, Bennett N, Aidman E, Nalivaiko E, Walker F (2021). Development of a modular stress management platform (Performance Edge VR) and a pilot efficacy trial of a bio-feedback enhanced training module for controlled breathing. PLoS One.

[37] Ladakis I, Kilintzis V, Xanthopoulou D, Chouvarda I (2021). Virtual Reality and Serious Games for Stress Reduction with Application in Work Environments. Proceedings of the 14th International Joint Conference on Biomedical Engineering Systems and Technologies.

[38] Mevlevioğlu D, Murphy D, Tabirca S (2021). Visual Respiratory Feedback in Virtual Reality Exposure Therapy: A Pilot Study. IMX '21: ACM International Conference on Interactive Media Experiences.

[39] Rockstroh C, Blum J, Göritz A (2020). A mobile VR-based respiratory biofeedback game to foster diaphragmatic breathing. Virtual Reality.

[40] van Rooij M, Lobel A, Harris O, Smit N, Granic I (2016). DEEP: A Biofeedback Virtual Reality Game for Children At-risk for Anxiety. CHI EA '16: Proceedings of the 2016 CHI Conference Extended Abstracts on Human Factors in Computing Systems.

[41] Brammer JC, van Peer JM, Michela A, van Rooij MMJW, Oostenveld R, Klumpers F, Dorrestijn W, Granic I, Roelofs K (2021). Breathing Biofeedback for Police Officers in a Stressful Virtual Environment: Challenges and Opportunities. Front Psychol.

[42] Rutkowski S, Rutkowska A, Kiper P, Jastrzebski D, Racheniuk H, Turolla A, Szczegielniak J, Casaburi R (2020). Virtual Reality Rehabilitation in Patients with Chronic Obstructive Pulmonary Disease: A Randomized Controlled Trial. Int J Chron Obstruct Pulmon Dis.

[43] Heng O, Albert Q (2020). Bubble Tower: Breathing Based Virtual Reality Action Game. BDIOT '20: Proceedings of the 2020 4th International Conference on Big Data and Internet of Things.

[44] Michela A, van Peer J, Brammer J, Nies A, van Rooij M, Oostenveld R, Dorrestijn W, Smit A, Roelofs K, Klumpers F, Granic I (2022). Deep-Breathing Biofeedback Trainability in a Virtual-Reality Action Game: A Single-Case Design Study With Police Trainers. Front Psychol.

[45] Gummidela V, Silva D, Gutierrez-Osuna R (2021). Evaluating the Role of Breathing Guidance on Game-Based Interventions for Relaxation Training. Front Digit Health.

[46] Tao Y Breath as an Input for VR Instrument. GitHub.

[47] Feinberg R, Lakshmi U, Golino M, Arriaga R (2022). ZenVR: Design Evaluation of a Virtual Reality Learning System for Meditation. CHI '22: Proceedings of the 2022 CHI Conference on Human Factors in Computing Systems.

[48] Tatzgern M, Domhardt M, Wolf M, Cenger M, Emsenhuber G, Dinic R, Gerner N, Hartl A (2022). AirRes Mask: A Precise and Robust Virtual Reality Breathing Interface Utilizing Breathing Resistance as Output Modality. CHI '22: Proceedings of the 2022 CHI Conference on Human Factors in Computing Systems.

[49] Betka S, Oliver K, Jemina F, Florian L, Sylvain C, Aline S, Thomas S, Paola S, Bruno H, Dan A, Olaf B (2022). Virtual reality intervention alleviates dyspnea in patients recovering from COVID pneumonia. European Respiratory Journal.

[50] Rodrigues IM, Lima AG, Santos AED, Santos ACA, Nascimento LSD, Serra MVCL, Pereira TDJS, Barbosa FDS, Seixas VM, Monte-Silva K, Scipioni KRDDS, Cruz DMCD, Piscitelli D, Goffredo M, Gois-Junior MB, Zanona ADF (2022). A Single Session of Virtual Reality Improved Tiredness, Shortness of Breath, Anxiety, Depression and Well-Being in Hospitalized Individuals with COVID-19: A Randomized Clinical Trial. J Pers Med.

[51] Bargas-Avila J, Hornbæk K (2011). Old wine in new bottles or novel challenges: a critical analysis of empirical studies of user experience. CHI '11: Proceedings of the SIGCHI Conference on Human Factors in Computing Systems.

[52] Deeks J, Higgins J, Altman D, Higgins J, Green S (2008). Analysing Data and Undertaking Meta-Analyses. Cochrane Handbook for Systematic Reviews of Interventions: Cochrane Book Series.

[53] NHS Centre for Reviews and Dissemination Undertaking systematic reviews of research on effectiveness: CRD's guidance for those carrying out or commissioning reviews. University of York.

[54] Faezipour M, Abuzneid A (2020). Smartphone-Based Self-Testing of COVID-19 Using Breathing Sounds. Telemed J E Health.

[55] Spielberger C (2010). State-Trait Anxiety Inventory. The Corsini Encyclopedia of Psychology.

[56] Spielberger C (1983). State-Trait Anxiety Inventory for Adults (STAI-AD). APA PsycTests.

[57] Spitzer RL, Kroenke K, Williams JBW, Löwe B (2006). A brief measure for assessing generalized anxiety disorder: the GAD-7. Arch Intern Med.

[58] Lee E (2012). Review of the psychometric evidence of the perceived stress scale. Asian Nurs Res (Korean Soc Nurs Sci).

[59] Ueshima J, Maeda K, Shimizu A, Nagano A, Ishida Y, Takeuchi T, Nonogaki T, Matsuyama R, Yamanaka Y, Murotani K, Mori N (2023). Cachexia staging score predicts survival in patients with cancer who receive palliative care. Nutrition.

[60] Papantoniou G, Moraitou D, Kaldrimidou M, Plakitsi K, Filippidou D, Katsadima E (2012). Affect and Cognitive Interference: An Examination of Their Effect on Self-Regulated Learning. Education Research International.

[61] Arevalo-Rodriguez I, Smailagic N, Roqué I Figuls M, Ciapponi A, Sanchez-Perez E, Giannakou A, Pedraza O, Bonfill Cosp X, Cullum S (2015). Mini-Mental State Examination (MMSE) for the detection of Alzheimer's disease and other dementias in people with mild cognitive impairment (MCI). Cochrane Database Syst Rev.

[62] Delgado DA, Lambert BS, Boutris N, McCulloch PC, Robbins AB, Moreno MR, Harris JD (2018). Validation of Digital Visual Analog Scale Pain Scoring With a Traditional Paper-based Visual Analog Scale in Adults. JAAOS Glob Res Rev.

[63] Lankoski P, Björk S (2015). Formal analysis of gameplay. Game Research Methods.

[64] Cowley B, Kosunen I, Lankoski P, Kivikangas J, Järvelä S, Ekman I, Kemppainen J, Ravaja N (2013). Experience Assessment and Design in the Analysis of Gameplay. Simulation & Gaming.

[65] Ruimi L, Hadash Y, Tanay G, Bernstein A State Mindfulness Scale (SMS). MindRxiv.

[66] IJsselsteijn W, Riva G, Riva G, Davide F, IJsselsteijn W (2003). Being There: The experience of presence in mediated environments. Being There: Concepts, effects and measurement of user presence in synthetic environments.

[67] Go Direct® Respiration Belt. Vernier.

[68] ZEISS VR One Plus Virtual Reality Smartphone Headset. B & H Foto & Electronics Corp.

[69] Polar H7 Heart Rate Sensor. Polar.

[70] Oculus Rift. Oculus.

[71] Polar H10 Heart Rate Sensor. Polar.

[72] BioHarness 3.0. Zephyr Technology.

[73] VIVE.

[74] NuvoAir spirometer. NuvoAir.

[75] Oculus Quest. Oculus.

[76] Huawei Nexus 6P specifications. GSMArena.

[77] Arduino Uno SMD board. Arduino.

[78] Wind Sensor Rev. C. Modern Device.

[79] ESP8266-01S - simple, easy to setup WiFi connectivity for Arduino. Nettigo.

[80] Adafruit Feather M0 Bluefruit LE. Adafruit.

[81] Equivital SEM (Sensor Electronics Module). ADInstruments.

[82] WristOx2® Model 3150 with USB. Nonin.

[83] PICO.

[84] Equivital. Philips Respironics.

[85] Oculus Rift S Features. Oculus.

[86] Rhythm+™ Wireless Fitness Heart Rate Monitor. Scosche.

[87] Moodmetric smart ring. Moodmetric.

[88] Oculus Go Standalone Virtual Reality Headset. Amazon.

[89] Wearable Sensor Products. Shimmer.

[90] Myndplay Myndband. MindTec Store.

[91] NVIDIA TITAN X. NVIDIA.

[92] Intel® Core™ i7-5820K Processor Specifications. Intel.

[93] Inductive Respiration (RIP) Sensor. PLUX Biosignals.

[94] Breathing+ Headset. Breathing Labs.

[95] Respiration Sensor - SA9311M. Thought Technology.

[96] Gear Sport. Samsung.

[97] Galaxy S9. Samsung.

[98] Gear VR. Samsung.

[99] Quest 2. Oculus.

[100] Oculos Realidade Virtual 3D Gamer Warrior - JS080. Arena Warrior.

[101] Explore VR TierOne. VR TierOne.

[102] NeXus-10 MKII. Mind Media.

[103] g.RESPsensor. Hooshmand Fanavar Tehran.

[104] Yeti - Premium Multi-Pattern USB Microphone with Blue VO!CE. Logitech G.

[105] ATSAMD21G18. Microchip Technology.

[106] AN-1181 – Using a MEMS Microphone in a 2-Wire Microphone Circuit. TDK InvenSense.

[107] SFM3300-D. Sensirion.

[108] 3M™ Reusable Full Face Mask 6000 Series. 3M.

[109] SERVO MOTOR SG90 Data Sheet. Imperial College London.

[110] Respiratory Effort Transducer, BSL | SS5LB. BIOPAC.

[111] B-Alert X10. iMotions.

[112] E4. Empatica.

[113] Hexoskin Smart Shirts. Hexoskin.

[114] Google.

[115] Nexus 4 smartphone. LG.

